# Comparative genomics of seven *Tenacibaculum maritimum* isolates from farmed Atlantic salmon (*Salmo salar*), Atlantic halibut (*Hippoglossus hippoglossus*), and lumpfish (*Cyclopterus lumpus*) in Canada and Norway reveals conserved features

**DOI:** 10.1371/journal.pone.0361557

**Published:** 2026-10-27

**Authors:** Erwan Lagadec, Snorre Gulla, Sverre Bang Småge, Enrique Pino-Martinez, Kjetil Solheim, Sigurd O. Handeland, Are Nylund

**Affiliations:** 1 Department of Biological Sciences, University of Bergen, Bergen, Norway; 2 Fish Health Research Group, Norwegian Veterinary Institute, Ås, Norway; 3 Cermaq Group AS, Oslo, Norway; 4 Sterling Halibut AS, Årdal i Ryfylke, Norway; Hokkaido University, JAPAN

## Abstract

*Tenacibaculum maritimum* is a major bacterial pathogen of marine fish responsible for ulcerative disease outbreaks and high mortality in numerous fish species and represents an important challenge in aquaculture worldwide. In this study, we characterized seven newly sequenced *T. maritimum* isolates recovered from farmed Atlantic salmon (*Salmo salar*), Atlantic halibut (*Hippoglossus hippoglossus*), and lumpfish (*Cyclopterus lumpus*) in British Columbia (Canada) and Norway, including the first complete genome of *T. maritimum* isolated from Atlantic salmon in Norway. Comparative genomic analysis revealed the presence of a complete Type IX secretion system (T9SS) and the canonical gliding-associated machinery. We also identified a largely conserved repertoire of candidate T9SS substrates and several major host-interaction proteins. Phylogenetic analysis placed the isolates in several well-supported clades comprising strains from different geographic origins and host species. Characterization of the O-antigen gene clusters identified three different types (1−0, 2−1, and 4−0), whose distribution was not congruent with the phylogenetic relationships among isolates. Colony development of the four Norwegian isolates on agar was strongly influenced by incubation temperature, with rapid growth at 20–24°C, slower growth at 12–16°C, and only limited growth at 8°C. This study expands the available genomic resources for *T. maritimum* and highlights the importance of integrating genomic and environmental surveillance to improve tenacibaculosis management in aquaculture systems.

## 1 Introduction

Tenacibaculosis is an ulcerative disease responsible for high mortalities in various marine fish species worldwide and is considered one of the most significant bacterial diseases for the aquaculture industry, resulting in major economic losses as well as impaired fish health and welfare [1].

*T. maritimum* was originally described as *Flexibacter maritimus* following its isolation from red seabream (*Pagrus major*) and gilthead (*Acanthopagrus schlegelii*) displaying eroded mouth, frayed fins and tail rot in Japan [2,3]. The bacterium was later reclassified as *T. maritimum* with the description of the genus *Tenacibaculum* in 2001 [4]. An extensive review of host distribution of *T. maritimum* isolates was presented by Avendaño-Herrera et al. [1] and was recently updated by Mabrok et al. [5].

*Tenacibaculum maritimum*, and the genus *Tenacibaculum* more generally, have long remained largely understudied. Although substantial knowledge gaps in *Tenacibaculum* diversity and pathogenicity persist [6], the rise in tenacibaculosis outbreaks in aquaculture has drawn attention to this major pathogen and driven increased research efforts. Detection and identification methods for *T. maritimum* have thus considerably improved over the years. Several PCR-based assays have been developed for its detection [7–13] and additional approaches such as fatty acid methylesters (FAMEs) profiling and MALDI-TOF mass spectrometry have been evaluated for isolate differentiation [14,15]. A multilocus sequence typing scheme developed by Habib et al. [16] has become a widely used typing method and has considerably enriched knowledge about the *Tenacibaculum* genus and *T. maritimum* in particular [17–20]. More recently, Lopez et al. [21] proposed a multiplex PCR-based serotyping scheme for genomic characterization of the *T. maritimum* O-antigen gene cluster (O-AGC).

The earliest known isolation of *T. maritimum* in Norway occurred in 2014 from kidneys of moribund farmed lumpfish (*Cyclopterus lumpus*) [18]. *T. maritimum* was subsequently isolated from farmed juvenile lumpfish again in 2016 [20] and 13 draft genomes of *T. maritimum* isolated from eight farmed Norwegian lumpfish and five turbot (*Scophthalmus maximus*) were released in 2024 [22]. Although present in Norwegian water, *T. maritimum* has until recently not been reported in association with ulcerative disease in Atlantic salmon (*Salmo salar*) in Norway. This contrasts with its documented role in “mouth rot” affecting farmed Atlantic salmon in British Columbia, Canada [23,24], and its association with ulcerative skin diseases in Atlantic salmon in Spain, Australia, and Chile [25–27]. In September 2024, *T. maritimum* was isolated from an ulcerative disease outbreak in a land-based Atlantic salmon facility in western Norway [28].

Previous comparative genomic analyses revealed a genetically structured *T. maritimum* population and indicated that several predicted virulence-associated factors are broadly conserved within the species [15]. However, genomic representation is uneven across host species and geographic regions. In particular, isolates from Norwegian Atlantic salmon displaying tenacibaculosis symptoms have not been represented.

In this study, we present genetic characterizations of seven newly sequenced *T. maritimum* isolates associated with disease to expand genomic representation across host species and geographic origins. Four isolates originated from Norway: two were isolated from Atlantic salmon, one from Atlantic halibut (*Hippoglossus hippoglossus*), and one from lumpfish. In addition, genomes of three isolates from Atlantic salmon in British Columbia, Canada, were included. The four Norwegian isolates were cultured at different incubation temperatures to examine colony development and in vitro response to temperature. This study provides the first complete genome of *T. maritimum* isolated from Atlantic salmon in Norway.

## 2 Materials and methods

### 2.1 Isolation

*T. maritimum* NLF15 was isolated in September 2015 from lethargic juvenile lumpfish displaying skin lesions in a fish facility in western Norway. Details about the outbreak and isolation are detailed in Småge et al. [20]. *T. maritimum* NH18 was recovered in September 2018 from farmed Atlantic halibut in the southwestern part of Norway. Mortality reached up to 10% in some sea cage units, and *T. maritimum* was isolated from the swollen gills of dead fish. *T. maritimum* NS17 was isolated in October 2017 from the jaw and gills of farmed Atlantic salmon in western Norway. The affected fish exhibited severe gill pathology, where several pathogens were suspected to be involved: *Paramoeba perurans*, *Paranucleospora theridion*, *Candidatus* Branchiomonas cysticola, and several *Tenacibaculum* species (*T. maritimum*, *T. dicentrarchi*, and *T. ovolyticum*). In September 2024, *T. maritimum* F23K6 was isolated from Atlantic salmon with ulcerative skin lesions reared in a land-based production facility in western Norway [28]. The three Canadian *T. maritimum* isolates CAN15−1, CAN16−1, and CAN16−2 were isolated from Atlantic salmon smolts showing clinical signs of mouthrot in saltwater farms in British Columbia, Canada, in September 2015, February 2016, and April 2016, respectively [19].

All isolates were preserved in 1:1 Biofreeze freezing medium (Biochrom™) and Marine broth (Difco 2216, BD Difco, Franklin Lakes, NJ, USA) in liquid nitrogen at the Fish Disease Research Group at the University of Bergen.

### 2.2 Whole genome sequencing and assembly

Genomic DNA from CAN15−1, CAN16−1, CAN16−2, NLF15, NS17, and NH18 isolates was extracted using the DNeasy Blood & Tissue kit (Qiagen, Hilden, Germany) according to the manufacturer’s pretreatment for Gram-negative bacteria. Short-read library preparation and sequencing were performed by BaseClear (Leiden, The Netherlands). Libraries were prepared using the Nextera XT DNA Library Preparation Kit (Illumina, San Diego, CA, USA). Paired-end reads of 2 x 150 bp were generated using an Illumina NovaSeq 6000 platform. FASTQ files were generated using bcl2fastq2 v2.18 [29]. Reads passing the Illumina Chastity filter were retained, reads containing PhiX control sequences were removed using a BaseClear in-house filtering protocol, and partial or complete adapter sequences were clipped, with a minimum retained read length of 50 bp. The quality of the processed reads was assessed using FastQC v0.11.5 [30]. Sequencing errors were corrected using BayesHammer [31] and corrected reads were assembled *de novo* using SPAdes version 3.10 [32]. Contigs were scaffolded using SSPACE v2.3 [33], and gaps were partially closed using GapFiller v1.10 [34]. The resulting draft assemblies were polished using Pilon v1.21 [35]. All sequences in the final assemblies were at least 300 bp long, and no additional filtering was performed by the authors.

Genomic DNA of *T. maritimum* isolate F23K6 was extracted using the Monarch HMW DNA Extraction Kit for Tissue (New England Biolabs, Ipswich, MA, USA) following the manufacturer’s High Molecular Weight DNA Extraction from Bacteria protocol. Library preparation and long-read sequencing were performed by Novogene (Cambridge, UK). A SMRTbell library was prepared using the SMRTbell Express Template Prep Kit 2.0 and sequencing was performed on a PacBio Sequel II platform in continuous long-read (CLR) mode (Pacific Biosciences, Menlo Park, CA, USA). The sequencing run generated 2.90 Gb distributed among 201,704 subreads, with a mean subread length of 14,443 bp and a subread N50 of 16,960 bp. *De novo* assembly was performed using Flye v2.9.6 [36] with the PacBio raw-read option (--pacbio-raw), an estimated genome size of 3.3 Mb, and a maximum initial assembly coverage of 200 × . Flye performed one internal polishing iteration and generated a single 3,390,651 bp contig with a mean coverage of 174× and classified the contig as circular. The circular contig was rotated in Geneious Prime v2025.2.1 (www.geneious.com) so that nucleotide 1 corresponded to the first nucleotide of the *dnaA* coding sequence.

Assembly statistics were calculated from all contigs using QUAST v5.3.0 [37], and genome completeness and contamination were estimated using CheckM2 v1.1.0 [38]. Details about the seven isolates, including host species, year of isolation, sequencing results, and sequence accession numbers are presented in Table 1.

**Table 1:** Origin details and genome assembly statistics for the seven *Tenacibaculum maritimum* isolates included in this study. F23K6 was sequenced using PacBio Sequel II (CLR) and assembled as a single contig. CAN15−1, CAN16−1, CAN16−2, NLF15, NS17, and NH18 draft genomes were generated using Illumina NovaSeq 6000 short-read sequencing.

Isolate	CAN15−1	CAN16−1	CAN16−2	NLF15	NS17	NH18	F23K6
Host	*Salmo salar*	*Salmo salar*	*Salmo salar*	*Cyclopterus lumpus*	*Salmo salar*	*Hippoglossus hippoglossus*	*Salmo salar*
Year	2015	2016	2016	2015	2017	2018	2024
Country	Canada	Canada	Canada	Norway	Norway	Norway	Norway
Sequencingplatform	Illumina NovaSeq 6000	Illumina NovaSeq 6000	Illumina NovaSeq 6000	Illumina NovaSeq 6000	Illumina NovaSeq 6000	Illumina NovaSeq 6000	PacBio Sequel II (CLR mode)
No of contigs	77	190	188	79	59	135	1
Assembly size (bp)	3,432,930	3,608,484	3,683,213	3,405,225	3,370,865	3,427,716	3,390,651
GC %	31.67	31.66	31.66	31.75	31.87	31.78	31.93
N50 (bp)	157,313	55,456	56,331	175,395	206,050	74,006	3,390,651
L50	6	22	21	7	6	16	1
Mean coverage (×)	177×	165×	162×	241×	211×	214×	174×
Completeness (%)	99.99	100.00	100.00	99.98	99.98	99.99	99.97
Contamination (%)	0.20	0.54	0.40	0.01	0.01	0.00	0.04
BioSample	SAMN54108665	SAMN54108666	SAMN54108667	SAMN49524046	SAMN49524047	SAMN49524045	SAMN52661613
SRA accession	SRR39816685	SRR39816684	SRR39816683	SRR39816681	SRR39816680	SRR39816682	SRR39816679

The genome assemblies and raw sequencing reads generated in this study have been deposited in NCBI under BioProject PRJNA1280491.

### 2.3 Genomic characteristics

#### 2.3.1 Visualization of the *Tenacibaculum maritimum* isolate F23K6 genome features.

The complete chromosome of *T. maritimum* isolate F23K6 (GenBank accession JBSXQI000000000) was visualized using Proksee [Build 2025-12-17] [39], with the circular genome map rendered using CGView v1.8.1 [40]. Coding DNA sequences, rRNA genes, tRNA genes were displayed using the default Proksee settings. GC content and GC skew were calculated using a 1-kb sliding window. Genomic islands were predicted using IslandViewer4 [41], which integrates sequence composition- and comparative genomics-based prediction methods (IslandPath-DIMOB, SIGI-HMM and IslandPick). Default parameters were used, and the predicted genomic island coordinates were visualized in Proksee. Type IX secretion system components and mobile element-associated genes were identified from eggNOG-mapper functional assignments, as described in the following section. Their genomic coordinates were extracted and imported into Proksee as custom annotation tracks.

#### 2.3.2 Comparative functional analyses.

For comparative genomics, all seven *T. maritimum* assemblies were annotated using Prokka v1.15.6 [42] using the same annotation workflow to ensure consistent gene prediction before pangenome reconstruction. Prokka was run using the bacterial kingdom setting and specifying *Tenacibaculum* as the genus and *maritimum* as the species and default settings otherwise. The resulting GFF files were used as input for Panaroo v1.5.1 [43] to reconstruct the pangenome and cluster orthologous genes. Panaroo was run in strict clean mode, with default settings otherwise. The resulting gene presence-absence matrix was used for downstream comparative analyses and integration with eggNOG-mapper functional annotations.

Protein sequences predicted by Prokka were functionally annotated using eggNOG-mapper v2.1.8 + galaxy4 [44] against eggNOG database v5.0.2 on the Galaxy Europe platform (https://usegalaxy.eu). Sequence searches were performed with DIAMOND in sensitive mode using the BLOSUM50 substitution matrix, gap-opening and gap-extension penalties of 13 and 3, respectively, an E-value threshold of 1 × 10^−5^ and minimum query and subject coverage thresholds of 50%. Seed orthologues were selected using an E-value threshold of 0.001 and the one-to-one orthology restriction. Panaroo gene-family assignments were linked to eggNOG-mapper annotations by genome and protein identifier. The merged dataset was used for comparative functional annotation of the seven genomes.

Type IX secretion systems were identified with MacSyFinder v2.1.6 [45] using the TXSScan v1.1.4 model collection [46]. The complete circular chromosome of F23K6 was analysed as an ordered circular replicon, whereas the six draft-genome proteomes were analysed as unordered datasets to account for assembly fragmentation. T9SS completeness was assessed from the TXSScan mandatory-component complement and system-wholeness score. Gliding-motility-associated proteins, including GldA–GldN, SprA, SprE and SprB-like proteins, were identified from the combined Panaroo and eggNOG-mapper annotations and evaluated for gene presence and predicted protein length.

Candidate T9SS substrates were identified by searching the seven proteomes with HMMER v3.4 [47] against the NCBI PGAP profile-HMMs TIGR04183.1 and TIGR04131.1, corresponding to Type A and Type B T9SS C-terminal sorting domains, respectively. Searches were performed using each model’s curated gathering threshold (--cut_ga), and the position of each detected domain relative to the protein C terminus was recorded. N-terminal signal peptides were predicted using DeepSig v1.2.5 [48] in Gram-negative bacterial mode. Proteins matching T9SS C-terminal-domain model were classified as candidate T9SS substrates, while the presence of a DeepSig-predicted N-terminal signal peptide was treated as additional supporting evidence.

Selected candidate virulence-associated proteins were identified by comparison with previously described proteins from the *T. maritimum* NCIMB 2154 reference genome (GenBank accession LT634361): CslA chondroitinase-AC (MARIT_2107), Sph sphingomyelinase (MARIT_1748), SiaA multimodular sialidase (MARIT_2686), an M43-family collagenase (MARIT_1085), and tenacilysin (MARIT_0124). Reference proteins were searched against the seven Prokka proteomes using BLASTP v2.17.0+ [49] with low-complexity filtering disabled and an E-value threshold of 1 × 10^-20^. Homologues were considered full-length when they showed at least 50% amino-acid identity and 90% query coverage. Protein lengths and adjacent partial matches were examined to identify potentially fragmented or split coding sequences.

Antimicrobial-resistance determinants were screened using ABRicate v1.2.0 (Seemann, ABRicate software repository) against the CARD [50] and NCBI AMR [51] databases, both updated on 17 December 2025. The genome assemblies were independently screened using NCBI AMRFinderPlus v4.2.5 [51] with database version 2025-12-03.1. Plasmids were screened using MOB-suite [52]. Prophage regions were identified using PHASTEST [53].

Genomic characterization of O-antigen gene clusters (O-AGCs) was performed *in silico* based on the multiplex PCR-based serotyping scheme developed by Lopez et al. [21]. For comparative visualization, annotated O-AGCs were analyzed by performing pairwise protein similarity searches and homologous genes were clustered based on sequence identity using clinker v0.0.32 [54].

### 2.4 *Tenacibaculum maritimum* phylogenies

In addition to the seven genomes generated as described above, 51 selected *T. maritimum* genome assemblies were retrieved from the NCBI RefSeq repository and included in the phylogenetic analyses. All genome assemblies with a valid RefSeq accession on 4 November 2025 were included with one exception: to reduce overrepresentation of densely sampled related populations, only a representative subset of assemblies from New Zealand and Tasmania were included (S1 Table). Core-gene and core-genome phylogenies were reconstructed using the ALPPACA pipeline v2.2.3 [55]. The core-gene track used Bakta v1.10.4 [56] with the v5 database and Panaroo v1.5.1 [43] in ‘sensitive’ mode. The core-genome track used ParSNP v2.1.3 [57], Gubbins v3.4 [58], and Maskrc-svg v0.5 [59]. Pairwise SNP distances for the core-gene analysis were calculated from the Panaroo core-gene alignment of the 58 genomes using snp-dists v0.8.2 (Seemann, snp-dists software repository, https://github.com/tseeman/snp-dists). Mean inter-clade and inter-distances were calculated as the mean of all pairwise SNP distances between isolates assigned to different groups. For both tracks, SNP-sites v2.5.1 [60] was used to identify variable sites, and maximum-likelihood phylogenies were inferred using IQ-TREE v2.4.0 [61]. ModelFinder [62] was used to select the best-fitting evolutionary model, and branch support was assessed using 10 000 ultrafast bootstrap iterations [63]. Default settings were otherwise used in both pipelines. Resulting phylogenies were midpoint-rooted for visualization.

### 2.5 Comparative growth of Norwegian *T. maritimum* isolates at different temperatures

The ability of the four Norwegian *T. maritimum* isolates to grow at different temperatures was assessed on agar plates. Isolates were recovered from nitrogen stocks on BAMA agar medium [64] and incubated at 20°C for 48 hours. For each isolate, a fresh colony was suspended in 20 mL of FMM broth (Condalab, Spain) in 250 mL Erlenmeyer and incubated 24 hours at 20°C with shaking at 230 rpm. Three aliquots of 20 µL of the bacterial culture were subsequently streaked onto three replicate BAMA agar plates and incubated at 4°C, 8°C, 12°C, 16°C, 20°C, and 24°C. Colony development was assessed visually and documented daily for seven days (plates at 4°C were observed for 14 days). β-hemolysis activity was assessed qualitatively on the blood-supplemented BAMA medium as previously described [64]. Plates were visually examined under a light source and β-hemolysis was identified as clearing or increased translucency of the blood agar extending beyond the bacterial growth. β-hemolysis could be distinguished from colony pigmentation (including the green-brown coloration after prolonged growth) which remained associated with the bacterial biomass. Expansion of the hemolytic zone was assessed qualitatively by comparing the visible clearing beyond the colony margin during successive daily observations. To determine bacterial concentration of the inoculum, ten-fold serial dilutions in FMM broth were spread on BAMA agar and incubated at 20°C until colonies were visible. Inoculum concentrations were 1.2x10^8^ (F23K6), 6.2x10^8^ (NLF15), 3.1x10^9^ (NS17), and 1.2x10^9^ (NH18) CFU/mL.

## 3 Results

### 3.1 Genome characteristics and comparative genomic features

Assembly of the *Tenacibaculum maritimum* isolate F23K6 genome using PacBio long reads resulted in a single circular chromosome of 3,390,651 bp with a GC content of 31.93% (Table 1). The chromosome contained 2,974 protein-coding sequences (CDSs) (Fig 1). Six complete *rrn* operons (5S – 16S – 23S) and 57 tRNA sequences were identified, consistent with previous *T. maritimum* full genome analysis [65,66]. The six Illumina assemblies comprised 59–190 contigs, with total assembly sizes ranging from 3,370,865 bp for NS17–3,683,213 bp for CAN16−2 and GC contents ranging from 31.66 to 31.87%. A single complete *rrn* operon was identified in each genome, likely due to the limitation of short-read assemblies to fully resolve multiple *rrn* operons. The number of predicted CDSs was consistent with previously sequenced *T. maritimum* genomes: 3,106 (CAN15−1); 3,530 (CAN16−1); 3,635 (CAN16−2); 3,059 (NLF15); 2,992 (NS17); and 3,098 (NH18). The comparatively high number of predicted CDSs in CAN16−1 and CAN16−2 were likely due to short-read assembly fragmentation and gene splitting across contig boundaries. Small fragment contigs may also contribute to the increased CDS counts. CheckM2-estimated assembly completeness ranged from 99.97% to 100.00%, and estimated contamination ranged from 0.00% to 0.54%.

**Fig 1:**
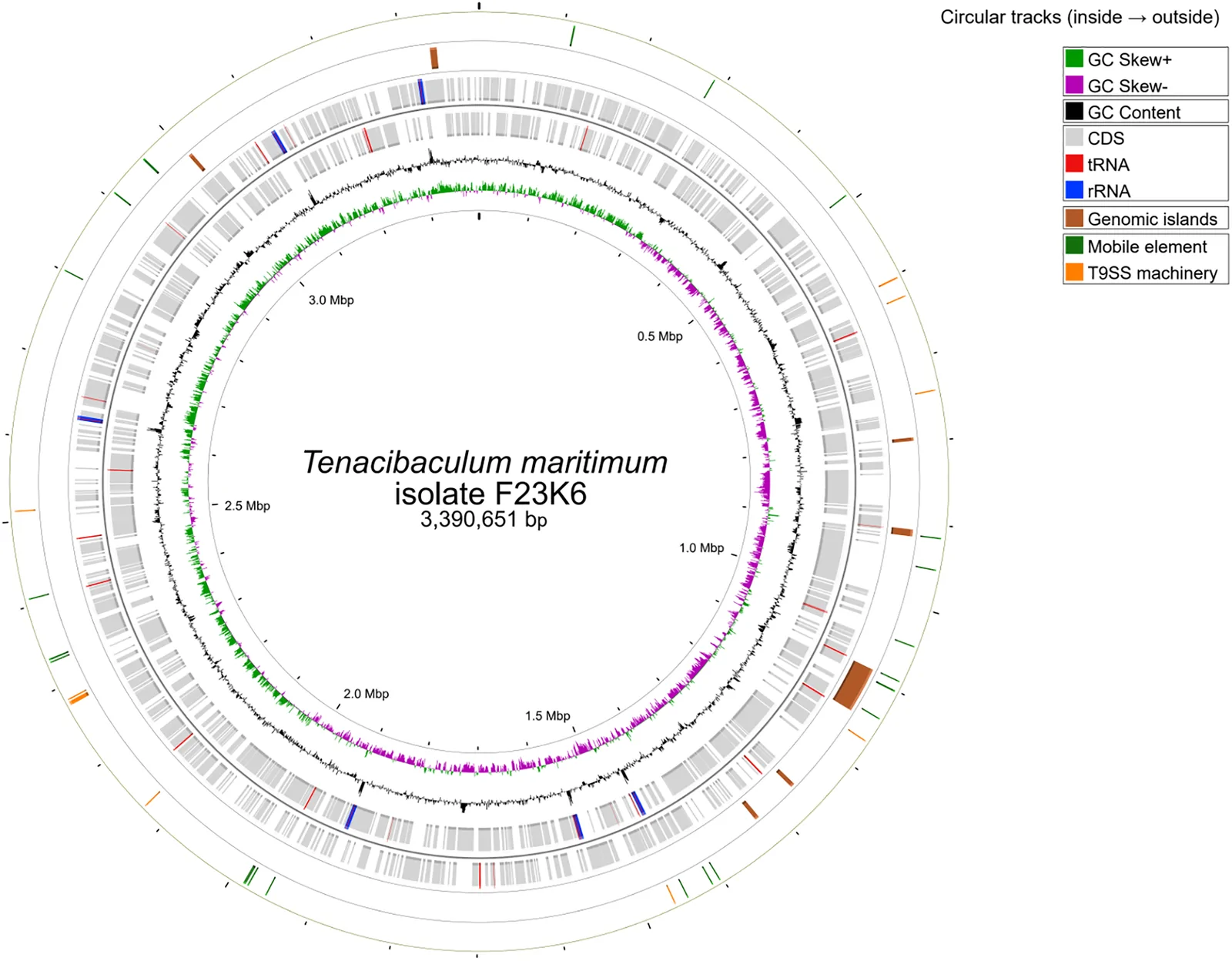
Circular genome map of *Tenacibaculum maritimum* isolate F23K6 generated using Proksee. From inner to outer tracks: GC skew (+ in green, – in pink); GC content sliding window; protein-coding sequences (in grey), rRNA (in blue) and tRNA (in red) on two rings (forward/reverse strands); genomic islands (in brown); mobile elements (in green) and T9SS machinery (in orange) on the outer single track.

Pangenome analysis of the seven consistently annotated genomes identified 4,775 orthologous gene families, including 2,312 core families present in all seven isolates, 1,640 shell families, and 823 cloud families. Because six genomes were draft assemblies, some accessory families may represent fragmented genes split across contig boundaries.

TXSScan identified a complete T9SS component repertoire in all seven isolates, with a system-wholeness score of 1.0 in each genome. Genes encoding the canonical gliding-associated proteins examined in this study, including GldA–GldN, SprA, SprE, PorT/SprT, and a major SprB-like protein, were also detected in all isolates. Screening for T9SS C-terminal sorting domains identified 49–53 candidate substrates per genome, comprising 42–45 proteins with Type A domains and 7–8 with Type B domains (Fig 2). Most candidate substrate families were shared among the seven isolates, indicating a broadly similar predicted T9SS cargo repertoire. The substrate analysis identified 359 CTD-positive proteins grouped into 60 candidate clusters, of which 44 were detected in all seven genomes (S2 Table).

**Fig 2:**
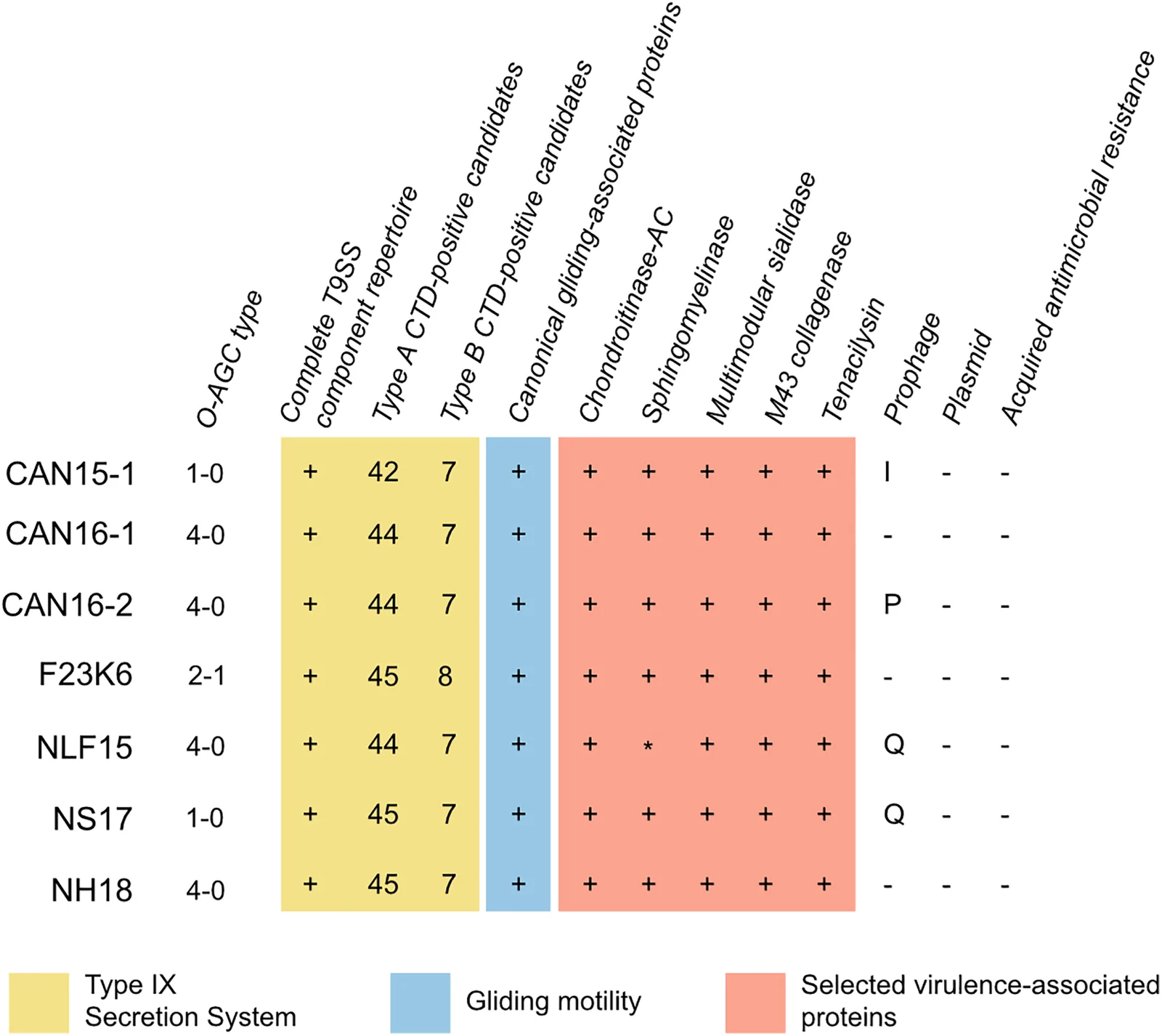
Comparative distribution of selected genomic features among the seven *Tenacibaculum maritimum* genomes. O-antigen gene-cluster types (O-AGC) were assigned using the multiplex PCR-based classification of Lopez et al. [21]. Type IX secretion system completeness was assessed with MacSyFinder v2.1.6 using the TXSScan v1.1.4 model collection. Candidate T9SS substrates correspond to proteins containing a Type A (TIGR04183.1) or Type B (TIGR04131.1) C-terminal sorting domain (CTD). The presence of canonical gliding-associated proteins was assessed from the combined Panaroo and eggNOG-mapper annotations. “+”: presence. “-“: not detected. “*”: split or uncertain (Sph homologous region in NLF15). Prophage regions predicted by PHASTEST are classified as intact (I), questionable (Q), partial/incomplete (P), or not detected (–). Numerical values indicate protein counts.

Reference-based protein comparisons detected homologues of five previously described candidate host-interaction proteins in all seven genomes: CslA chondroitinase-AC (CslA), sphingomyelinase (Sph), the multimodular sialidase (SiaA), an M43-family collagenase, and tenacilysin (Fig 2). Full-length homologues were identified in all isolates except for the Sph homologue in NLF15, which was represented by two adjacent predicted coding sequences corresponding to different portions of the reference protein. The conservation of these selected proteins and of the T9SS indicates limited variation in the genomic features examined.

No antimicrobial-resistance genes were detected in any of the seven genomes using ABRicate with either the CARD or NCBI AMR database, or using NCBI AMRFinderPlus. No plasmids were identified using MOB-suite. PHASTEST identified a 23,512-bp intact prophage region in CAN15−1, an 11,443-bp incomplete prophage region in CAN16−2, and identical 21,493-bp questionable prophage regions in NLF15 and NS17. No prophage regions were detected in CAN16−1, NH18, or F23K6 (S1 Fig). The O-antigen gene cluster was identified in all seven genomes by *in silico* PCR screening using primers developed by [21]. A 196 bp amplicon matching the P1F/R primer set was detected in isolates NS17 and CAN15−1, thereby assigning them to O-AGC type 1−0. Three amplicons matching P1 F/R (196 bp), P2/3 F/R (357 bp), and P6 F/R (476 bp) primer sets were detected in isolate F23K6, classifying F23K6 as O-AGC type 2−1. A 986 bp amplicon was generated with primer set P4 F/R in isolates NLF15, NH18, CAN16−1, and CAN16−2, assigning them to O-AGC type 4−0. A similar classification was supported using clinker (S2 Fig).

### 3.2 *Tenacibaculum maritimum* phylogenies

The core-gene phylogeny inferred using Panaroo and IQ-TREE, was based upon a 2.45 Mb alignment with 83,513 parsimonious sites and spanning 2216 core genes and was built under the GTR + F + I + R6 evolutionary model. It resolved the isolates into two main clades separated by an average of 39,460 SNPs and additional internal structuring (Fig 3). The seven isolates sequenced in this study did not form a single clade and were distributed among separate well-supported lineages. Most *T. maritimum* isolates included in the analysis clustered within one strongly supported major clade, including the Norwegian isolates NH18 and F23K6. This major clade was further divided into subclades A, B, and C as previously described [15,16,67], and each separated from one another by an average of 28,095 SNPs. Isolate F23K6 was most closely related to strain TM-KORJJ, isolated in South Korea from olive flounder (*Paralichthys olivaceus*), with both strains included in subclade B. NH18, isolated from Atlantic halibut, belonged to subclade C together with two Norwegian isolates from lumpfish and two Spanish isolates, one from Coho salmon (*Oncorhynchus kisutch*) and one from Atlantic salmon. Subclade A, on the other hand, contained exclusively three Mediterranean isolates.

**Fig 3:**
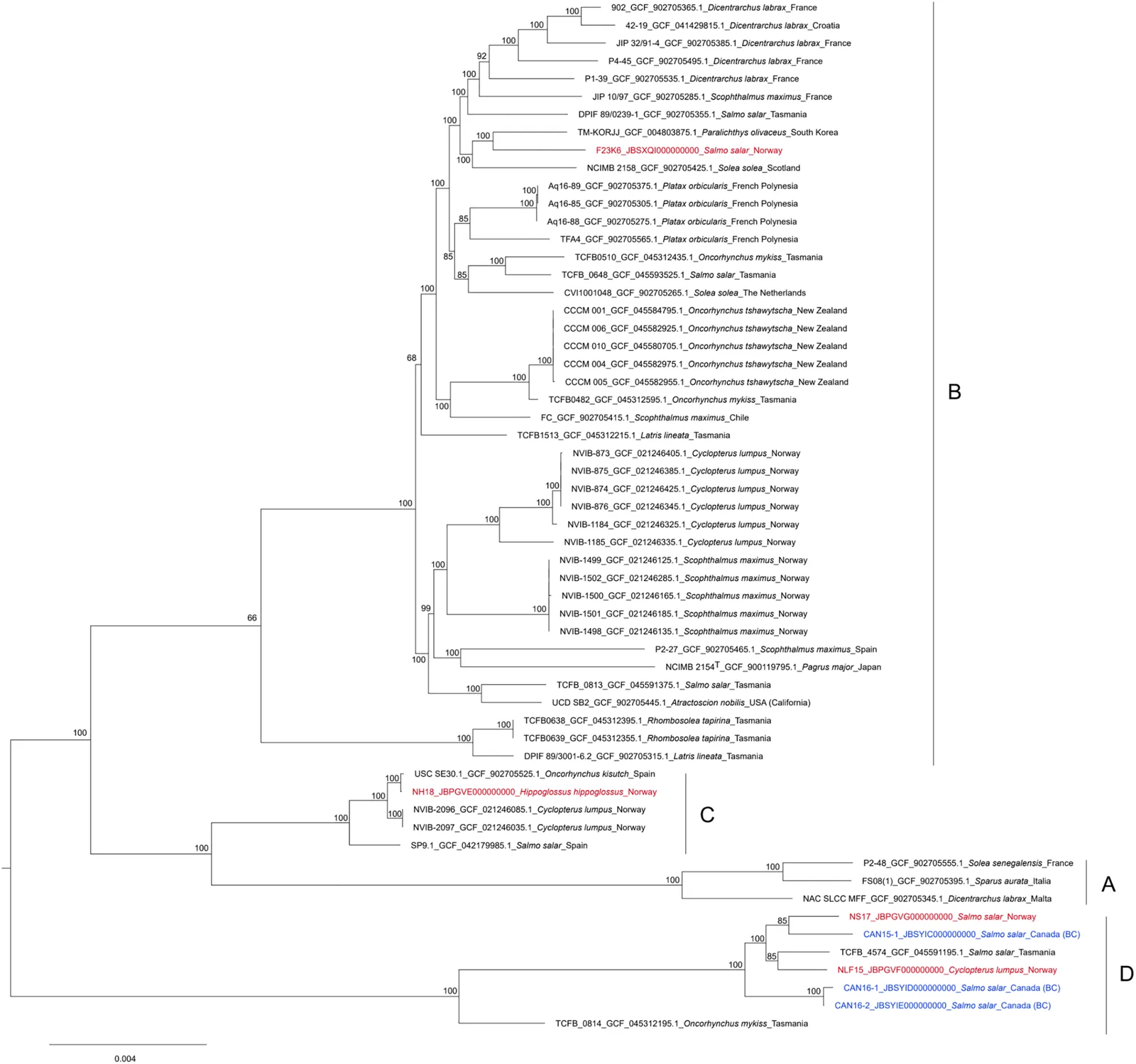
Core-gene phylogeny of *Tenacibaculum maritimum* genomes. Core-gene phylogeny inferred using Panaroo and IQ-TREE from a 2.45 Mb alignment of 51 *T. maritimum* genome assemblies from NCBI RefSeq and seven genomes generated in this study. Norwegian and Canadian isolates are highlighted in red and blue, respectively. Ultrafast bootstrap values are indicated at each node. Sequence labels include strain name, accession number, host species, and isolation origin. Clade designations A-C follow the core-genome nomenclature of [15]; clade D is a distinct clade provisionally designated.

The Canadian Atlantic salmon isolates CAN15−1, CAN16−1, and CAN16−2 formed a distinct, strongly supported monophyletic clade together with the Norwegian isolates NLF15 and NS17 and two Tasmanian *T. maritimum* isolates, TCFB 4574 obtained from Atlantic salmon and TCFB 0814 isolated from rainbow trout (*Oncorhynchus mykiss*). This clade is here provisionally described clade D.

Among the outbreak-associated *T. maritimum* isolates included in the present dataset, isolates originated from the same outbreak formed well-supported monophyletic clades. The isolates Aq16–89, Aq16–85, and Aq16–88, obtained during an outbreak in orbicular batfish (*Platax* *orbicularis*) in French Polynesia, clustered together. Similarly, isolates NVIB-1498, −1499, −1500, −1501, and −1502, obtained from turbot during an outbreak in Norway in 2016 formed a distinct cluster. The four isolates NVIB-873, −874, −875, and −876, obtained from lumpfish during a single outbreak in a southern production area in Norway [22], also formed a strongly supported clade. By contrast, NVIB-2096 and NVIB-2097, which were obtained from lumpfish during a separate outbreak in a distinct Norwegian production area, clustered together in a different clade.

The core-genome phylogeny was based upon a 2.46 Mb alignment with 65,420 parsimonious sites and was built under the GTR + F + I + R5 evolutionary model. It shows a topology and structure similar to the core-gene phylogeny (S3 Fig).

### 3.3 Growth of *T. maritimum* isolates at different temperatures

Qualitative colony development of the four Norwegian *T. maritimum* isolates varied strongly with incubation temperature. After 48 h, no visible growth was observed at 4°C and 8°C, while only slight growth was detected at 12°C. In contrast, growth was clearly visible at 16°C, and extensive growth along the inoculated streak was clearly established at 20°C and 24°C with well-developed colonies. Colonies grown at 16°C displayed a pale purple coloration and the characteristic rough surface morphology of *T. maritimum*, whereas more advanced colonies at 20°C and 24°C began to develop a darker green-brown coloration (Fig 4). Growth was already detectable after 24 h at 16°C, 20°C, and 24°C, but not at 8°C or 12°C. At 8°C, faint growth became visible only after approximately 5 days and remained limited after seven days. Growth at 12°C developed progressively over the incubation period, but colonies remained small and little apparent lateral spreading was observed. In contrast, at 20°C and 24°C, growth was already well established along the inoculated streak by 48 h. Little or no further lateral colony expansion was observed after this period. No visible growth was observed at 4°C after 14 days.

**Fig 4:**
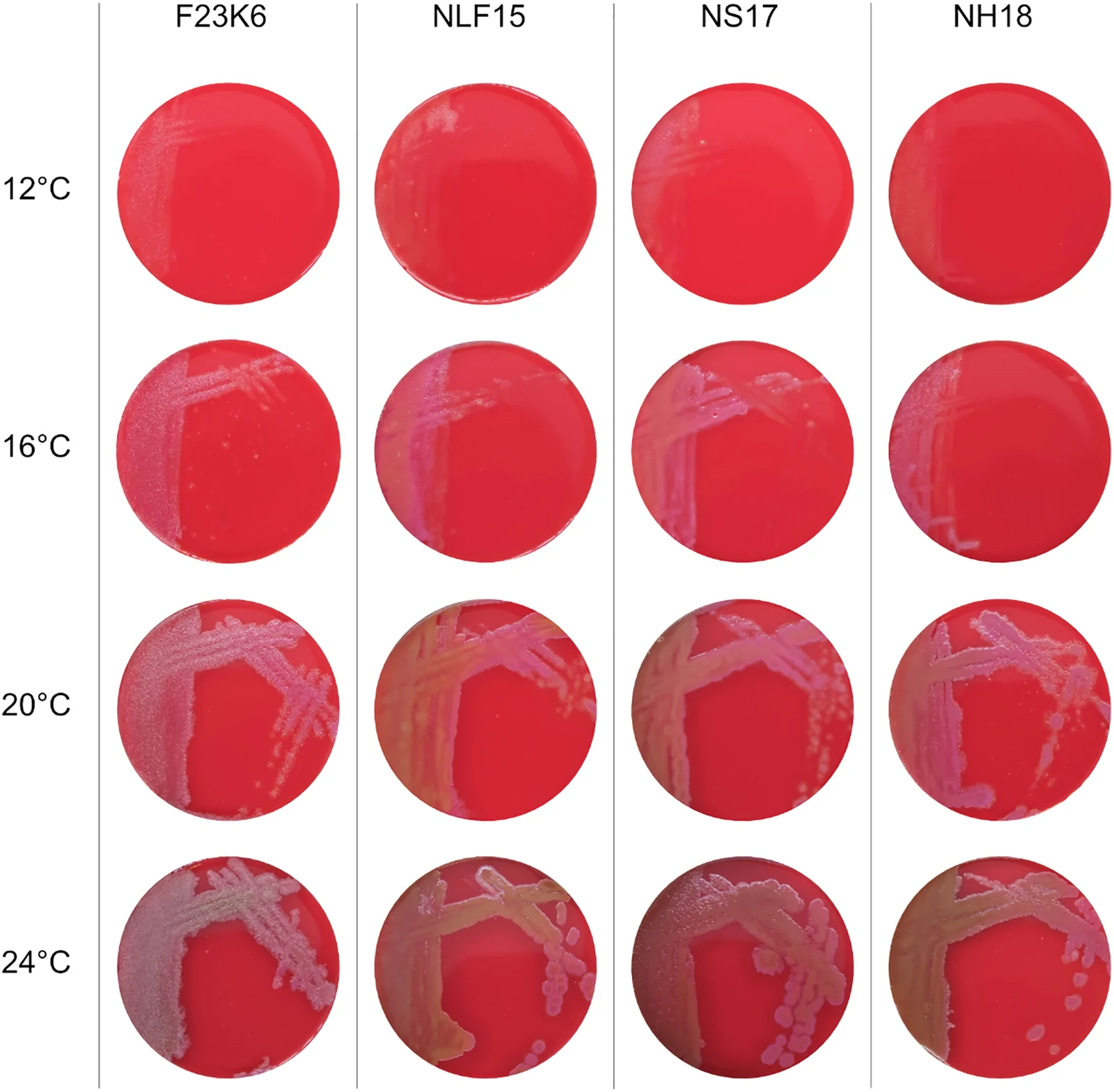
Qualitative growth of four Norwegian *Tenacibaculum maritimum* isolates at different temperatures. Growth of F23K6, NLF15, NS17, and NH18 on BAMA agar plates after 48 h of incubation at 12°C, 16°C, 20°C, and 24°C. Plates were inoculated by streaking 20 µL of bacterial culture containing 1.2x10^8^ (F23K6), 6.2x10^8^ (NLF15), 3.1x10^9^ (NS17), and 1.2x10^9^ (NH18) CFU/mL. One representative plate from three technical replicate plates is shown for each condition.

Qualitatively assessed β-hemolysis could be detected after 48 h at 16°C, 20°C, and 24°C and after approximately 4–5 days at 12°C, while no visible hemolysis was observed at 8°C. At 20 and 24°C, the hemolytic area appeared to extend progressively beyond the colony margin, even after no further colony spreading was apparent (S4 Fig). Hemolytic-zone dimensions were not quantified.

As the four inoculum concentrations differed substantially among isolates (from 1.2x10^8^ CFU/mL for F23K6 to 1.2x10^9^ CFU/mL for NH18), bacterial growth was not compared quantitatively between the four isolates. Comparable qualitative patterns were observed on the three replicate agar plates for each condition.

## 4 Discussion

Seven *Tenacibaculum maritimum* isolates recovered during outbreaks in farmed Atlantic salmon, Atlantic halibut, and lumpfish, in Norway and Canada (British Columbia) were sent for whole genome sequencing. This study expands the genomic resources available for this major fish pathogen and includes the first complete genome of *T. maritimum* isolated from Atlantic salmon in Norway.

Despite their different host and geographic origins, these isolates shared a broadly conserved repertoire of secretion, motility, and putative host-interaction features. Bridel et al. [15] previously showed that genomes of 25 *T. maritimum* isolates from diverse hosts and geographic origins contain several major predicted virulence-associated factors as well as T9SS components. The present results are consistent with it and extend these observations to the seven additional isolates from Norway and Canada. Comparative genomics of functional gene categories revealed that virulence-associated systems are also conserved across the seven *T. maritimum* genomes, including a complete Type IX secretion system (T9SS) and the gliding-motility machinery. The predicted T9SS cargo was also broadly similar. The curated substrate inventory included several putative extracellular peptidases, carbohydrate-active proteins and major gliding adhesins. These are known components of the virulence process including surface attachment, degradation of extracellular substrates, invasion, colonization and destruction of host tissue, and biofilm formation [66,68]. The conservation of CslA, Sph, SiaA, the M43-family collagenase, and tenacilysin further suggests that several mechanisms potentially involved in tissue degradation and membrane damage are widely distributed within the species. Their presence across isolates from different hosts and phylogenetic lineages is consistent with the broad host range of *T. maritimum*. While genomic plasticity is supported by minor variations in accessory components (such as prophage acquisition, mobile genetic elements), the apparent absence of isolate-specific gain or loss of major virulence factors suggests that the genetic component associated with host interaction is largely conserved within the species. However, conservation of these genes does not demonstrate gene expression or protein activity among isolates.

Previous core-genome phylogenetic reconstructions supported subdivision of the *T. maritimum* population into three main clades A, B, and C [15]. More recently, Rudenko et al. [67] described two divergent Tasmanian lineages C1 and C2, based on the earlier MLST-based clade C classification [16]. This nomenclature does not match directly with the core-genome clades of Bridel et al. [15]. Notably, TCFB 0518 (= DPIF 89/300-6.2) used by Rudenko et al. [67] to link C1 to the MLST-based clade C, was assigned to core-genome clade B by Bridel et al. [15] Furthermore, clade A was not represented in the dataset from Rudenko et al. [67]. In the broader dataset presented here, the previously described core-genome clades A-B-C were recovered with DPIF 89/300-6.2 remaining in clade B, while USC SP9.1 and USC SE30.1 remaining in clade C. In contrast, TCFB 4574 and TCFB 0814, assigned to C2 by Rudenko et al. [67], formed a separate strongly supported clade together with the three isolates from Canada (CAN15−1, CAN16−1, CAN16−2), and NS17 and NLF15 from Norway. We therefore retain the core-genome A_C nomenclature of Bridel et al. [15] and provisionally designate this additional clade as clade D. The high congruence between core-genome and core-gene phylogenies, which respectively did and did not involve recombination filtering, suggests that homologous recombination did not substantially affect the placement of individual sub-lineages for this particular dataset.

Overall, both phylogenies reveal a highly structured *T. maritimum* population, but without clear patterns according to host species or geographic origin. Isolates from the same host species or region are distributed across multiple phylogenetic clades, which is consistent with previous observations [15,16,22], and suggests that *T. maritimum* does not exhibit a strong degree of host specificity or local endemism. However, a different pattern was apparent at the scale of individual outbreaks. In the present dataset, isolates recovered from the same outbreak formed well-supported monophyletic group, which is consistent with previously proposed model of tenacibaculosis outbreaks associated with *T. maritimum* occurring as local, clonal or near-clonal epizootics [1,69]. This is notably in contrast to the commonly observed multi-strain involvement during *T. finnmarkense*-associated tenacibaculosis outbreaks in farmed Atlantic salmon [70], suggesting distinct epidemiological dynamics among pathogenic *Tenacibaculum* species.

Genome analyses also revealed that the O-antigen gene clusters (O-AGCs) displayed three main organizational structures corresponding to types 1−0 (NS17, CAN15−1), 2−1 (F23K6), and 4−0 (NLF15, NH18, CAN16−1, CAN16−2), previously described by Lopez et al. [21]. Early studies hypothesized that *T. maritimum* O-serotypes were host-specific [71]. However, several recent studies have shown that this is not necessarily the case [21,72–74], and the present results further confirm this observation. Two different O-AGC types (1−0 and 4−0) were identified among isolates recovered from a single host species (Atlantic salmon) within the same region (British Columbia, Canada). Moreover, irrespective of geographic origin, O-AGC types 1−0, 2−1, and 4−0 were identified in *T. maritimum* isolates from Atlantic salmon. *T. maritimum* O-AGC classification was not congruent with the core-gene phylogeny, and O-AGCs of the same type did not consistently cluster within one monophyletic clade. This suggests that the loci involved in the O-antigen biosynthesis evolve independently from the core genome through horizontal gene transfer or recombination as already suggested [21], and that surface antigen variation may be driven by selective pressures (e.g., host immune interactions or environmental adaptation). The availability of several new genomes belonging to O-AGC type 4 may facilitate future investigations into mitigation strategies against this emerging O-AGC type such as strain selection for vaccine development.

*T. maritimum* is generally regarded as a mesophilic bacterium that can grow at temperatures ranging from approximately 15–34°C [1,4]. Increased prevalence and severity of tenacibaculosis have likewise been reported at higher temperature, particularly above 15 °C [1,69,75,76]. This likely contributes to the observed seasonality of the disease, with outbreaks occurring preferentially during summer and autumn or extended period of elevated seawater temperatures [26,72,77,78]. Seawater temperatures in Norway are generally low, which may partly explain the historically limited impact of *T. maritimum* in this region. However, climate change and associated ocean warming may favor bacterial persistence and increase the risk of future outbreaks. In 2023, the Norwegian Fish Health Report, issued annually by the Norwegian Veterinary Institute, reported detections of *T. maritimum* in wrasse at three different locations, and in Atlantic salmon at a single location in western Norway [79]. The following years, *T. maritimum* was reported from 15 and 17 Atlantic salmon farms distributed along the southern and western Norwegian coast in 2024 and 2025, respectively [80,81]. These findings were based on compiled data from the Norwegian Veterinary Institute and private laboratories, but as tenacibaculosis is not a notifiable fish disease in Norway, the total number of annual occurrences may have been higher.

In this context, *T. maritimum* NH18 was isolated from an Atlantic halibut production facility in 2018 after seawater temperature had remained above 16°C for more than a month. Similarly, F23K6 was isolated from a land-based Atlantic salmon facility in western Norway in 2024 during a period when seawater temperature exceeded 15°C [82]. Consistent with the temperature conditions associated with these outbreaks, qualitative colony development of the four Norwegian isolates from this study was strongly temperature dependent in vitro. After 48 h on BAMA agar plates, no visible growth was observed at 4°C or 8°C, only very slight growth was detected at 12°C, and growth was clearly established at 16°C and extensive along the inoculated streak at 20°C and 24°C. These observations demonstrate that elevated temperature markedly accelerated colony development in all four isolates under the BAMA agar conditions. Moreover, visually assessed β-hemolysis also became apparent earlier at the higher incubation temperatures, with extension beyond the visible colony margin during subsequent observations and continued to expand after lateral colony growth had stopped. This pattern suggests that production, release, and activity of extracellular membrane-damaging compounds may also be temperature dependent. Several proteins detected in the genomes, including tenacilysin and sphingomyelinase, may be involved in such activity. However, neither hemolysin production nor extracellular protein abundance or activity was measured, therefore the mechanism underlying the observed hemolytic activity cannot be determined from this experiment. The ability of the Norwegian isolates to grow slowly at 8°C may permit persistence during cooler periods, whereas rapid growth at temperatures above approximately 16°C could facilitate seasonal increases in bacterial abundance. However, whether these *T. maritimum* isolates could persist in aquaculture environments during cooler periods remains unknown. The higher prevalence of *T. maritimum* outbreaks during periods of high seawater temperatures suggests that temperature is an important environmental driver of *T. maritimum* proliferation, but this remains an ecological hypothesis that requires field-based studies. Longitudinal monitoring of seawater temperature, bacterial abundance, and disease development would be important for following the distribution and impact of *T. maritimum* in Norwegian aquaculture.

Overall, this study supports the model in which *T. maritimum* is a widely distributed marine bacterium with a conserved pathogenic potential. From an applied perspective, the limited genomic variation observed between isolates suggests that predicting outbreak risk and severity based solely on genotyping may be insufficient. The marked effect of temperature on bacterial growth under the tested conditions supports temperature as a potential major environmental factor for *T. maritimum* ecology and highlights the need for environmental monitoring. Continued surveillance of the presence of *T. maritimum* remains essential in aquaculture systems, including on facility surfaces where the bacterium may form profuse biofilms that facilitate environmental persistence [68,82,83].

## Supporting information

S1 Table Publicly available *Tenacibaculum maritimum* genome assemblies included in the phylogenetic analyses. Metadata were obtained from the corresponding NCBI RefSeq/BioSample records. NA: not available in the NCBI record.
(XLSX)

S2 Table Candidate Type IX secretion system substrates containing Type A or Type B C-terminal sorting domains (CTD) in seven *Tenacibaculum maritimum* isolates. Candidate substrates were identified with HMMER v3.4 using the NCBI PGAP profile HMMs TIGR04183.1 (Type A CTD) and TIGR04131.1 (Type B CTD). The dataset corresponds to 60 candidate families (50 Type A and 10 Type B), representing 359 retained proteins. Curated predicted functions were manually assigned.
(XLSX)

S1 Fig Identification and annotations of prophage regions in *Tenacibaculum maritimum* isolates CAN15−1, CAN16−2, NLF15, and NS17 using PHASTEST (www.phastest.ca). (PDF)

S2 Fig Comparative organization of the O-antigen gene cluster (O-AGC) in the seven *Tenacibaculum maritimum* isolates described in this study. Arrows represent transcriptional orientation of predicted coding sequences. Homologous genes shared between isolates are shown in the same color.
(PDF)

S3 Fig Core-genome phylogeny of *Tenacibaculum maritimum* genomes. Core-genome phylogeny generated via ParSNP and IQ-TREE based upon a 2.46 Mb alignment of 51 *T. maritimum* genome assemblies from NCBI RefSeq and seven genomes generated in this study. Norwegian and Canadian isolates are highlighted in red and blue, respectively. Ultrafast bootstrap values are indicated at each node. Sequence labels include strain name, accession number, host species, and isolation origin.
(PDF)

S4 Fig Growth of four Norwegian *Tenacibaculum maritimum* isolates on BAMA agar plates at 4°C, 8°C, 12°C, 16°C, 20°C, and 24°C over a period of seven days. A) F23K6, B) NLF15, C) NS17, D) NH18. Representative plates from three technical replicates for each temperature are shown after 24, 48, 72, 120, and 168h of growth. Plates incubated at 4°C are not shown because no visible growth was observed after 14 days. Arrowheads indicate representative regions of blood-supplemented agar clearing interpreted as β-hemolytic activity.
(PDF)
