# Supplementary material for: Comparative genomics of seven Tenacibaculum maritimum isolates from farmed Atlantic salmon (Salmo salar), Atlantic halibut (Hippoglossus hippoglossus), and lumpfish (Cyclopterus lumpus) in Canada and Norway reveals conserved features
Source: PLoS One. 2026 Oct 27;21(10):e0361557. doi: 10.1371/journal.pone.0361557 (PMC13712720; doi:10.1371/journal.pone.0361557)
Supplement: Supplementary file 1 [file pone.0361557.s003.pdf]

Prophage sequences within *Tenacibaculum maritimum* strains CAN15-1, CAN16-2, NLF15, and NS17. Criteria for scoring prophage regions (as intact, questionable, or incomplete) and identification by Phastest (www.phastest.ca) (Wishart et al. 2023)

Strain	Contig	Location (bp)	Size (bp)	GC content	Completeness (score)	Predicted phage
CAN15-1	contig_00001	49236-72747	23512	33.85%	Intact (110)	PHAGE_Flavob_vB_FspM_pippi8_1_NC_048830
CAN16-2	contig_00002	2857-14299	11443	29.95%	Incomplete (50)	PHAGE_Flavob_vB_FspM_lotta8_1_NC_048829
NLF15	contig_00002	131710-153202	21493	31.15%	Questionable (80)	PHAGE_Flavob_vB_FspM_lotta8_1_NC_048829
NS17	contig_00007	61899-83391	21493	31.15%	Questionable (80)	PHAGE_Flavob_vB_FspM_lotta8_1_NC_048829

***Tenacibaculum maritimum* strain CAN15-1, contig_00001**

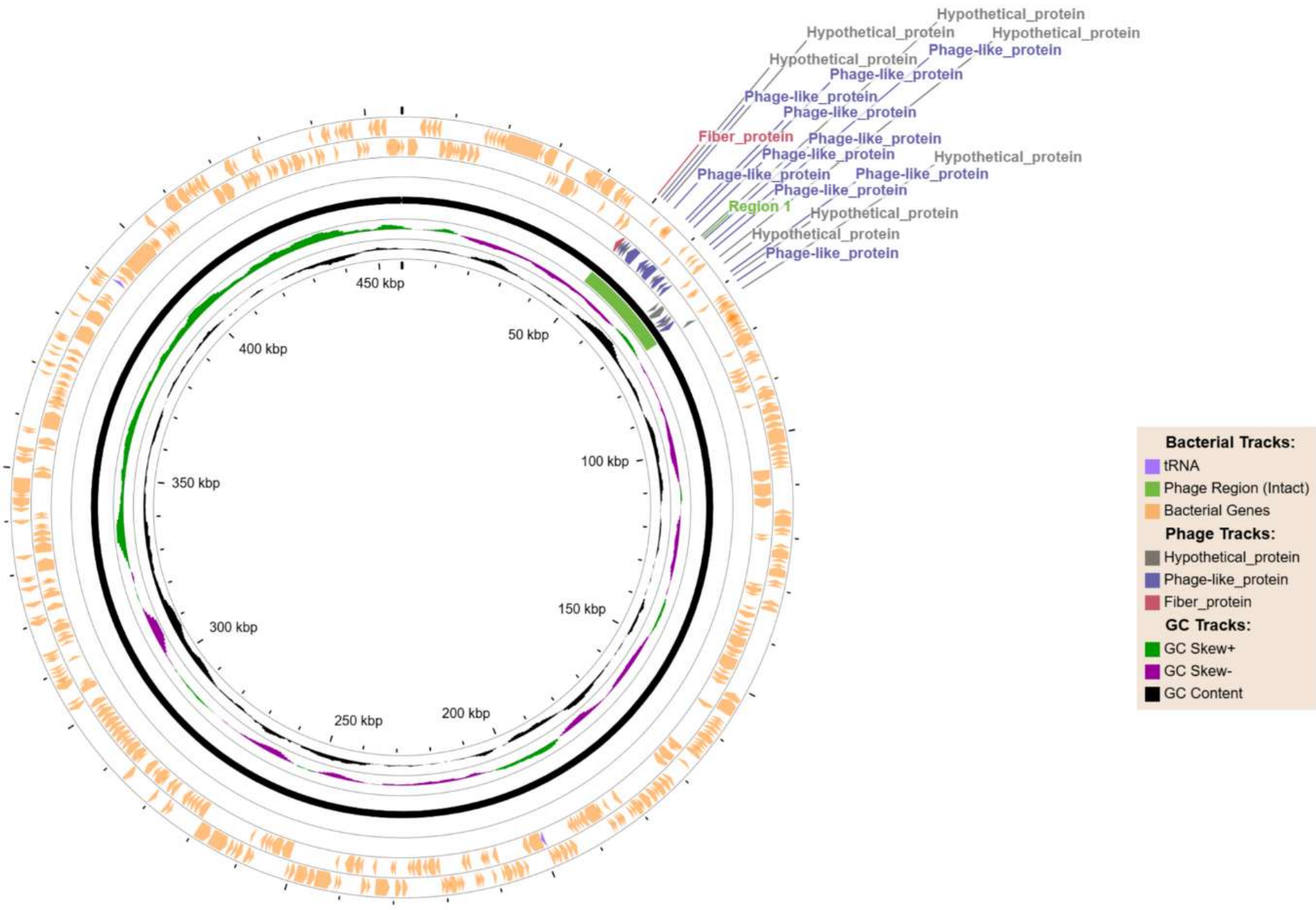

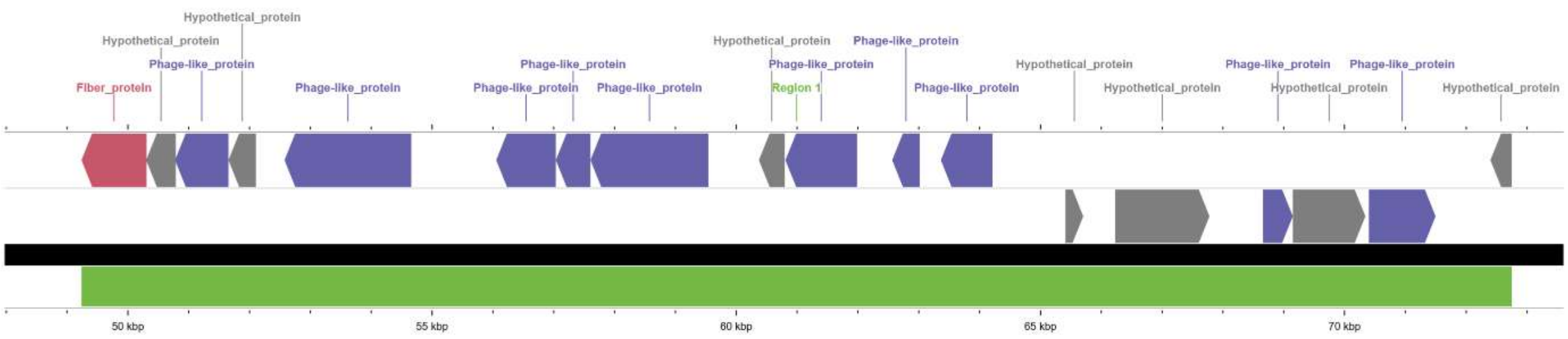

Tenacibaculum maritimum strain CAN16-2, contig_00002

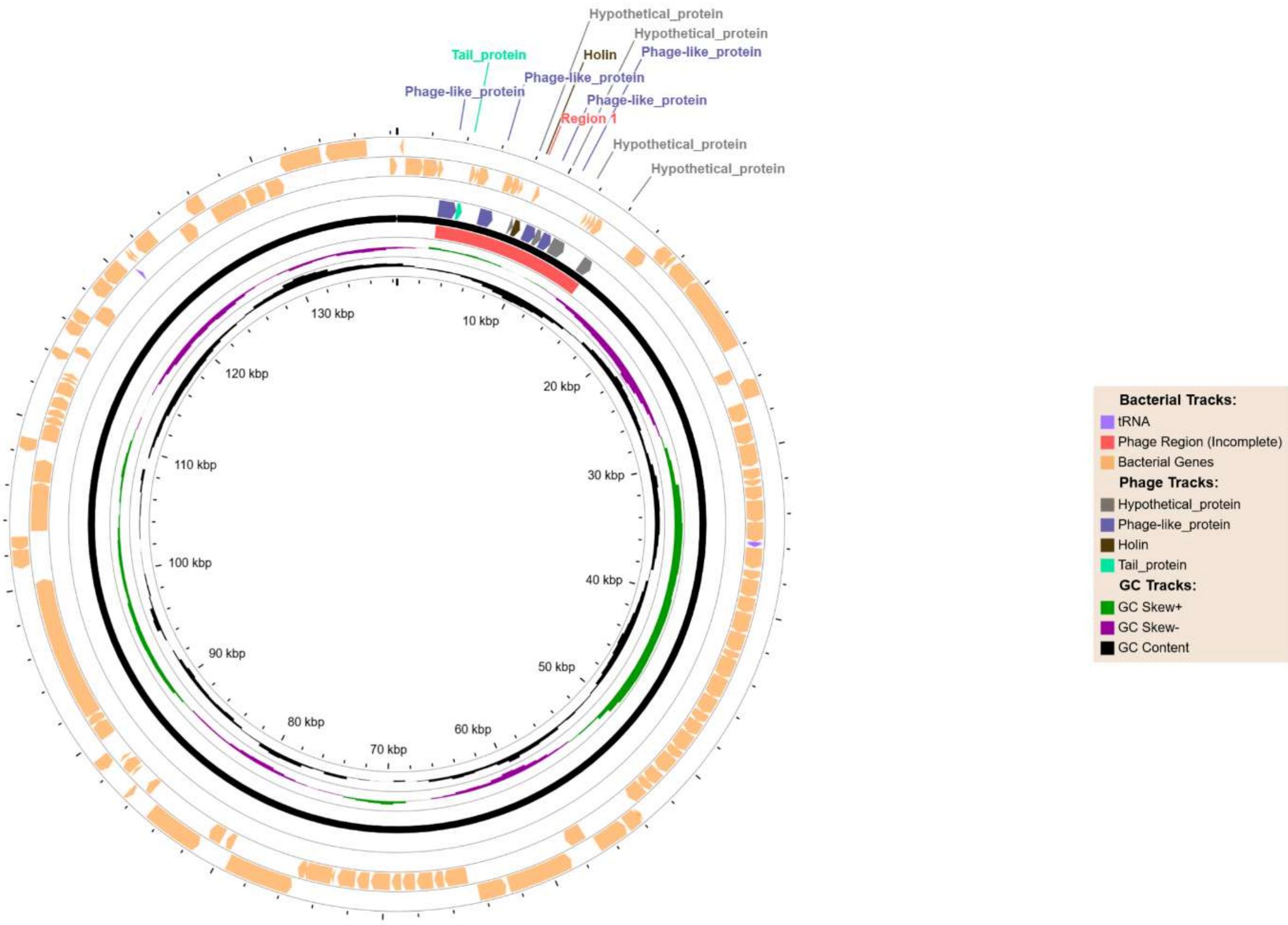

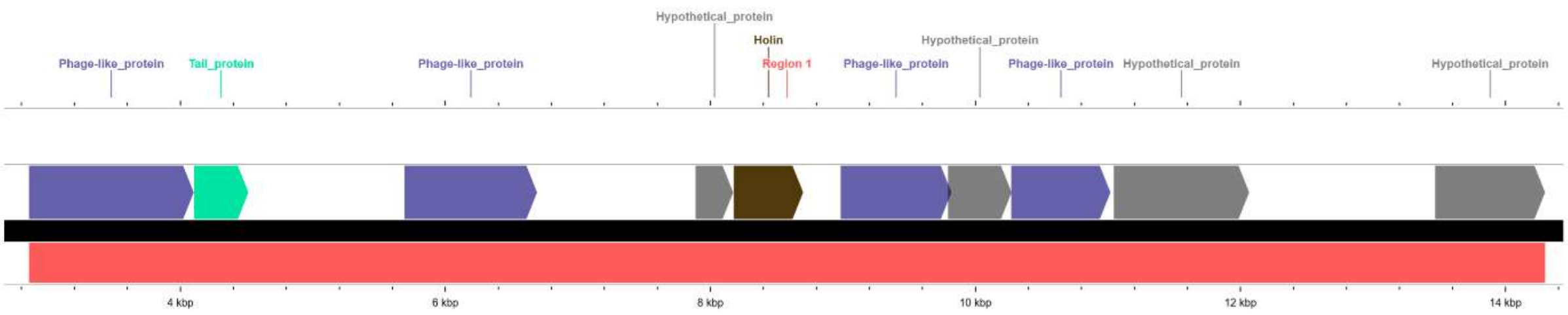

Tenacibaculum maritimum strain NLF15, contig_00002

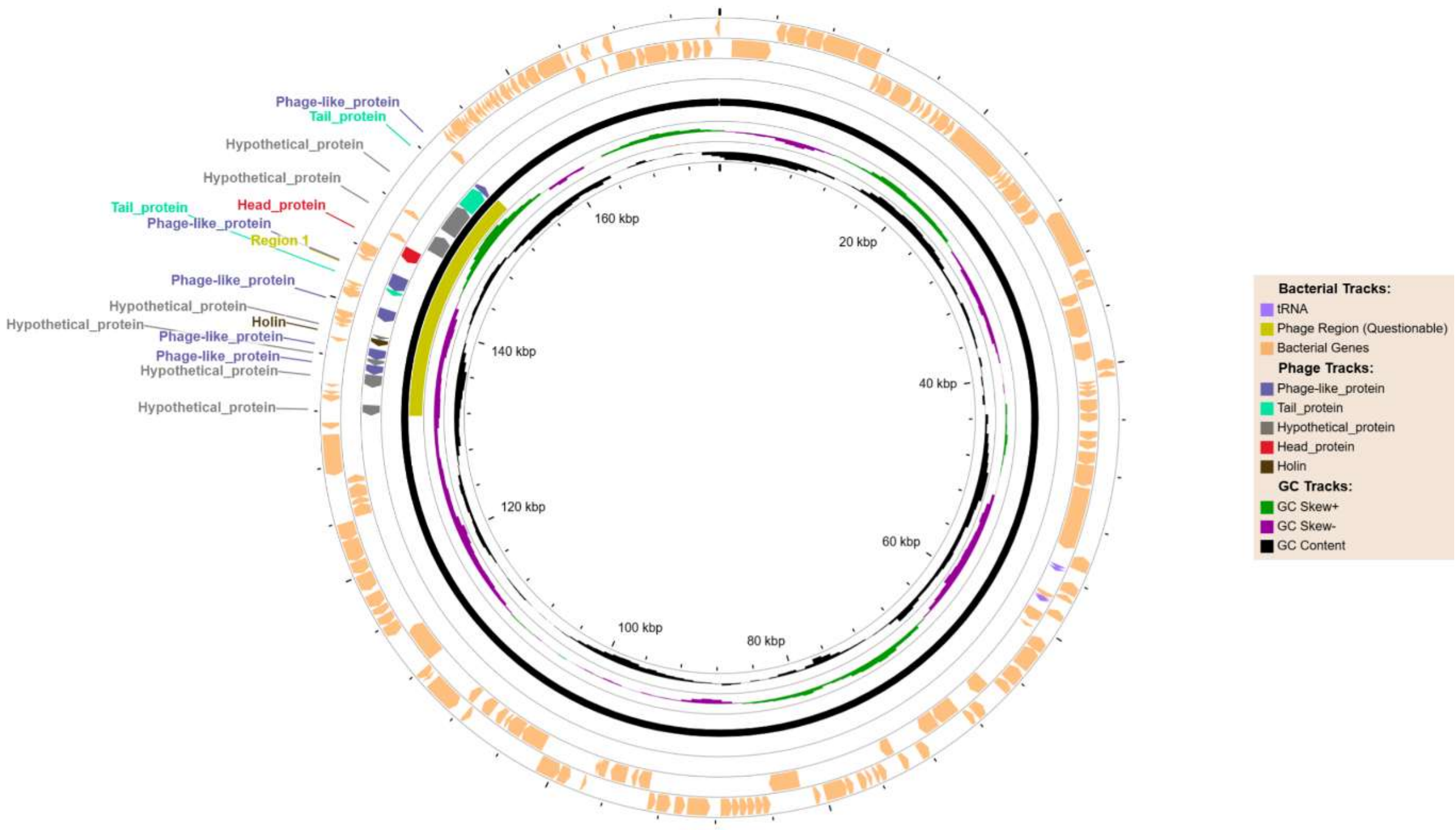

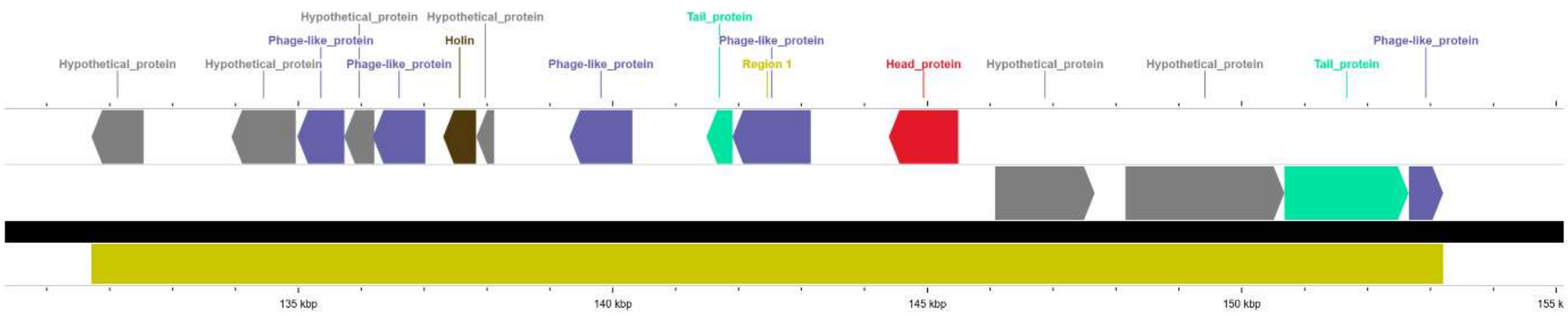

***Tenacibaculum maritimum* strain NS17, contig_00007**

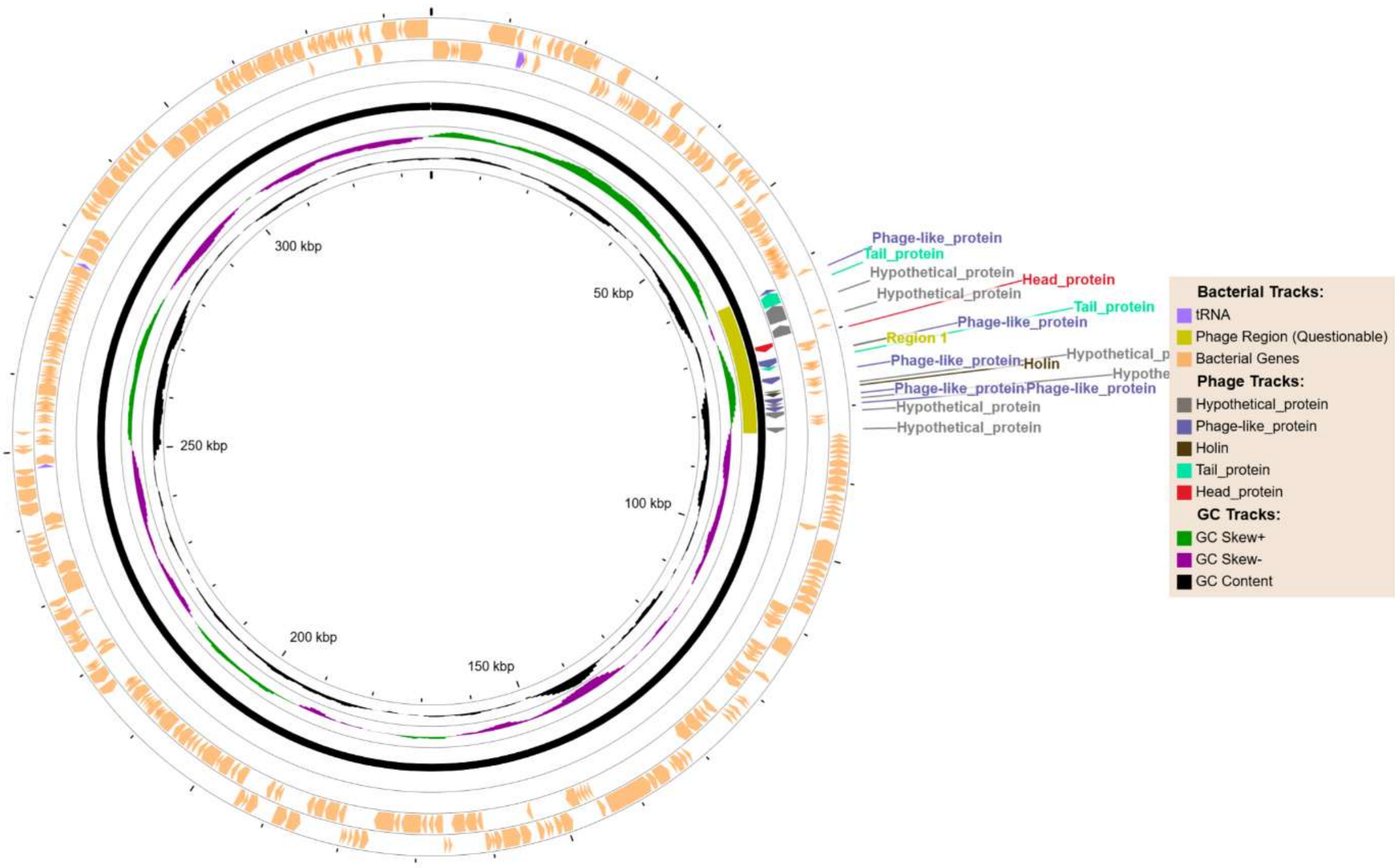

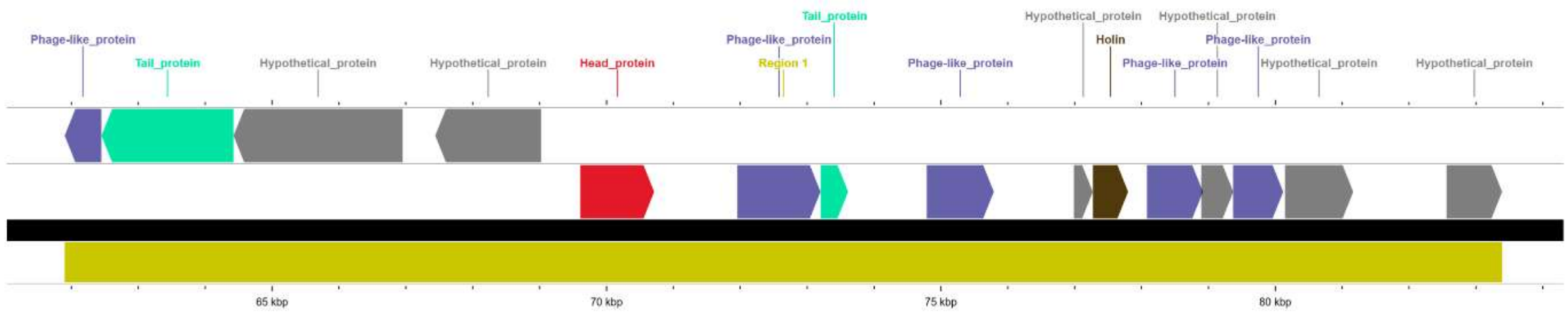

Reference

Wishart, D. S., S. Han, S. Saha, E. Oler, H. Peters, J. R. Grant, P. Stothard, and V. Gautam. 2023. 'PHASTEST: faster than PHASTER, better than PHAST', *Nucleic Acids Res*, 51: W443-w50.
