# Supplementary material for: Comparative genomics of seven Tenacibaculum maritimum isolates from farmed Atlantic salmon (Salmo salar), Atlantic halibut (Hippoglossus hippoglossus), and lumpfish (Cyclopterus lumpus) in Canada and Norway reveals conserved features
Source: PLoS One. 2026 Oct 27;21(10):e0361557. doi: 10.1371/journal.pone.0361557 (PMC13712720; doi:10.1371/journal.pone.0361557)
Supplement: Supplementary file 2 [file pone.0361557.s004.pdf]

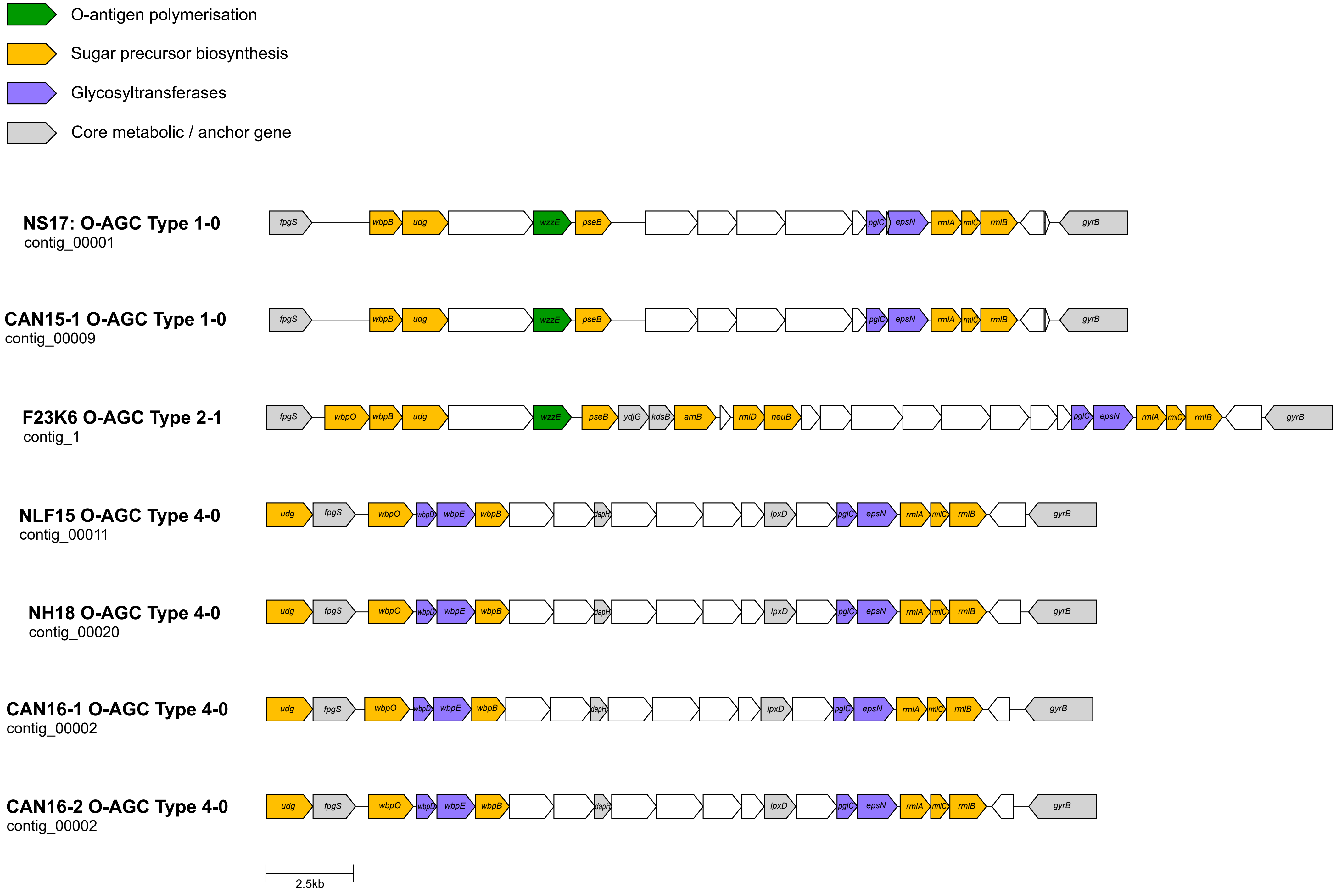
O-antigen polymerisation
Sugar precursor biosynthesis
Glycosyltransferases
Core metabolic / anchor gene
NS17: O-AGC Type 1-0
contig_00001
CAN15-1 O-AGC Type 1-0
contig_00009
F23K6 O-AGC Type 2-1
contig_1
NLF15 O-AGC Type 4-0
contig_00011
NH18 O-AGC Type 4-0
contig_00020
CAN16-1 O-AGC Type 4-0
contig_00002
CAN16-2 O-AGC Type 4-0
contig_00002
fpgS
wbpB
udg
wzzE
pseB
pglC
epsN
rmlA
rmlC
rmlB
gyrB
wbpO
ydjG
kdsB
arnB
rmlD
neuB
wbpD
wbpE
dapH
lpxD
2.5kb
