# Supplementary material for: Comparative genomics of seven Tenacibaculum maritimum isolates from farmed Atlantic salmon (Salmo salar), Atlantic halibut (Hippoglossus hippoglossus), and lumpfish (Cyclopterus lumpus) in Canada and Norway reveals conserved features
Source: PLoS One. 2026 Oct 27;21(10):e0361557. doi: 10.1371/journal.pone.0361557 (PMC13712720; doi:10.1371/journal.pone.0361557)
Supplement: Supplementary file 3 [file pone.0361557.s005.pdf]

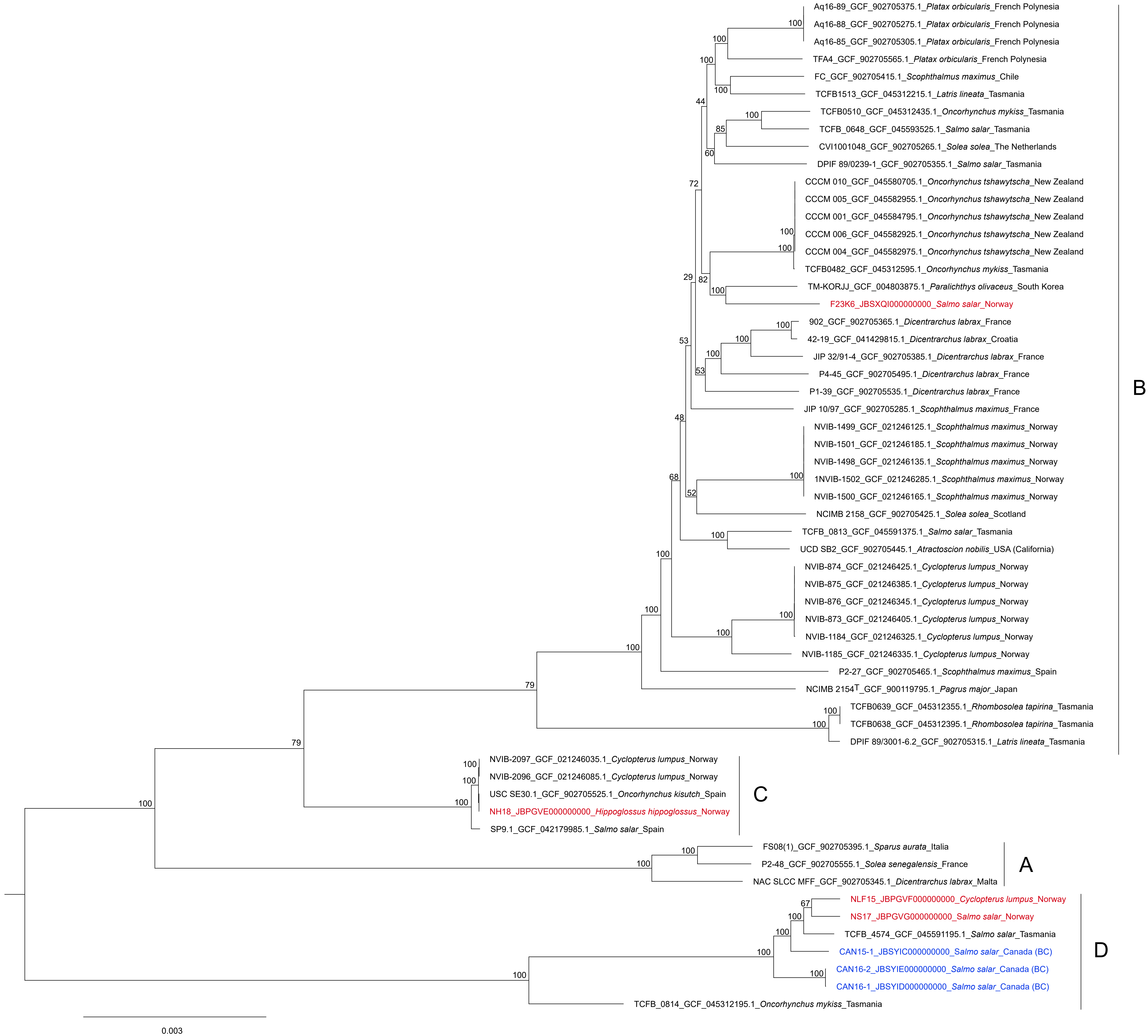

Aq16-89_GCF_902705375.1_*Platax orbicularis*_French Polynesia
Aq16-88_GCF_902705275.1_*Platax orbicularis*_French Polynesia
Aq16-85_GCF_902705305.1_*Platax orbicularis*_French Polynesia
TFA4_GCF_902705565.1_*Platax orbicularis*_French Polynesia
FC_GCF_902705415.1_*Scophthalmus maximus*_Chile
TCFB1513_GCF_045312215.1_*Latris lineata*_Tasmania
TCFB0510_GCF_045312435.1_*Oncorhynchus mykiss*_Tasmania
TCFB_0648_GCF_045593525.1_*Salmo salar*_Tasmania
CVI1001048_GCF_902705265.1_*Solea solea*_The Netherlands
DPIF 89/0239-1_GCF_902705355.1_*Salmo salar*_Tasmania
CCCM 010_GCF_045580705.1_*Oncorhynchus tshawytscha*_New Zealand
CCCM 005_GCF_045582955.1_*Oncorhynchus tshawytscha*_New Zealand
CCCM 001_GCF_045584795.1_*Oncorhynchus tshawytscha*_New Zealand
CCCM 006_GCF_045582925.1_*Oncorhynchus tshawytscha*_New Zealand
CCCM 004_GCF_045582975.1_*Oncorhynchus tshawytscha*_New Zealand
TCFB0482_GCF_045312595.1_*Oncorhynchus mykiss*_Tasmania
TM-KORJJ_GCF_004803875.1_*Paralichthys olivaceus*_South Korea
F23K6_JBSXQI000000000_*Salmo salar*_Norway
902_GCF_902705365.1_*Dicentrarchus labrax*_France
42-19_GCF_041429815.1_*Dicentrarchus labrax*_Croatia
JIP 32/91-4_GCF_902705385.1_*Dicentrarchus labrax*_France
P4-45_GCF_902705495.1_*Dicentrarchus labrax*_France
P1-39_GCF_902705535.1_*Dicentrarchus labrax*_France
JIP 10/97_GCF_902705285.1_*Scophthalmus maximus*_France
NVIB-1499_GCF_021246125.1_*Scophthalmus maximus*_Norway
NVIB-1501_GCF_021246185.1_*Scophthalmus maximus*_Norway
NVIB-1498_GCF_021246135.1_*Scophthalmus maximus*_Norway
1NVIB-1502_GCF_021246285.1_*Scophthalmus maximus*_Norway
NVIB-1500_GCF_021246165.1_*Scophthalmus maximus*_Norway
NCIMB 2158_GCF_902705425.1_*Solea solea*_Scotland
TCFB_0813_GCF_045591375.1_*Salmo salar*_Tasmania
UCD SB2_GCF_902705445.1_*Atractoscion nobilis*_USA (California)
NVIB-874_GCF_021246425.1_*Cyclopterus lumpus*_Norway
NVIB-875_GCF_021246385.1_*Cyclopterus lumpus*_Norway
NVIB-876_GCF_021246345.1_*Cyclopterus lumpus*_Norway
NVIB-873_GCF_021246405.1_*Cyclopterus lumpus*_Norway
NVIB-1184_GCF_021246325.1_*Cyclopterus lumpus*_Norway
NVIB-1185_GCF_021246335.1_*Cyclopterus lumpus*_Norway
P2-27_GCF_902705465.1_*Scophthalmus maximus*_Spain
NCIMB 2154T_GCF_900119795.1_*Pagrus major*_Japan
TCFB0639_GCF_045312355.1_*Rhombosolea tapirina*_Tasmania
TCFB0638_GCF_045312395.1_*Rhombosolea tapirina*_Tasmania
DPIF 89/3001-6.2_GCF_902705315.1_*Latris lineata*_Tasmania
B
NVIB-2097_GCF_021246035.1_*Cyclopterus lumpus*_Norway
NVIB-2096_GCF_021246085.1_*Cyclopterus lumpus*_Norway
USC SE30.1_GCF_902705525.1_*Oncorhynchus kisutch*_Spain
NH18_JBPGVE000000000_*Hippoglossus hippoglossus*_Norway
SP9.1_GCF_042179985.1_*Salmo salar*_Spain
C
FS08(1)_GCF_902705395.1_*Sparus aurata*_Italia
P2-48_GCF_902705555.1_*Solea senegalensis*_France
NAC SLCC MFF_GCF_902705345.1_*Dicentrarchus labrax*_Malta
A
NLF15_JBPGVF000000000_*Cyclopterus lumpus*_Norway
NS17_JBPGVG000000000_*Salmo salar*_Norway
TCFB_4574_GCF_045591195.1_*Salmo salar*_Tasmania
CAN15-1_JBSYIC000000000_*Salmo salar*_Canada (BC)
CAN16-2_JBSYIE000000000_*Salmo salar*_Canada (BC)
CAN16-1_JBSYID000000000_*Salmo salar*_Canada (BC)
TCFB_0814_GCF_045312195.1_*Oncorhynchus mykiss*_Tasmania
D
0.003
