# Supplementary material for: Comparative genomics of seven Tenacibaculum maritimum isolates from farmed Atlantic salmon (Salmo salar), Atlantic halibut (Hippoglossus hippoglossus), and lumpfish (Cyclopterus lumpus) in Canada and Norway reveals conserved features
Source: PLoS One. 2026 Oct 27;21(10):e0361557. doi: 10.1371/journal.pone.0361557 (PMC13712720; doi:10.1371/journal.pone.0361557)

A. Growth of F23K6 on BAMA agar plate

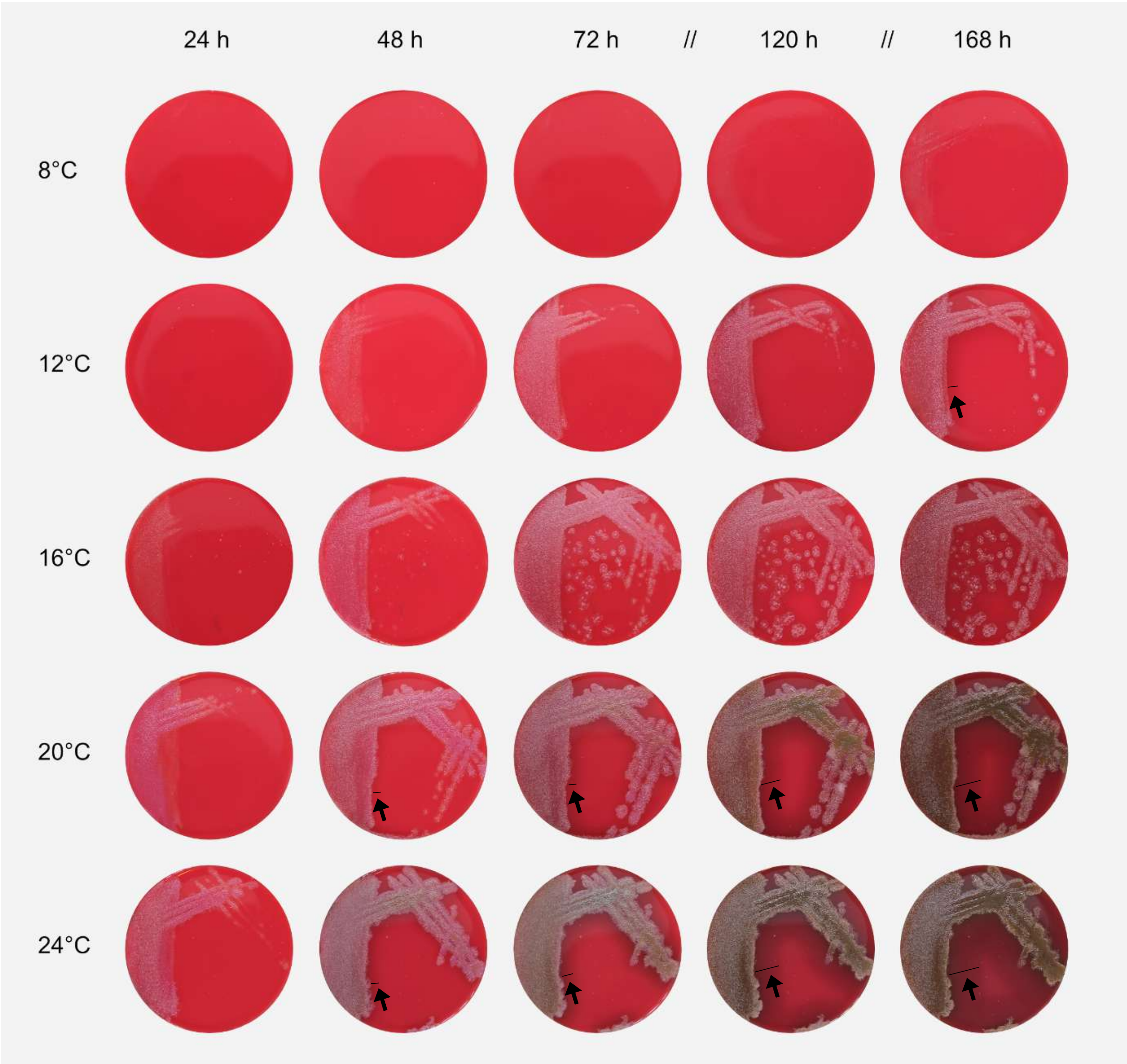

B. Growth of NLF15 on BAMA agar plate

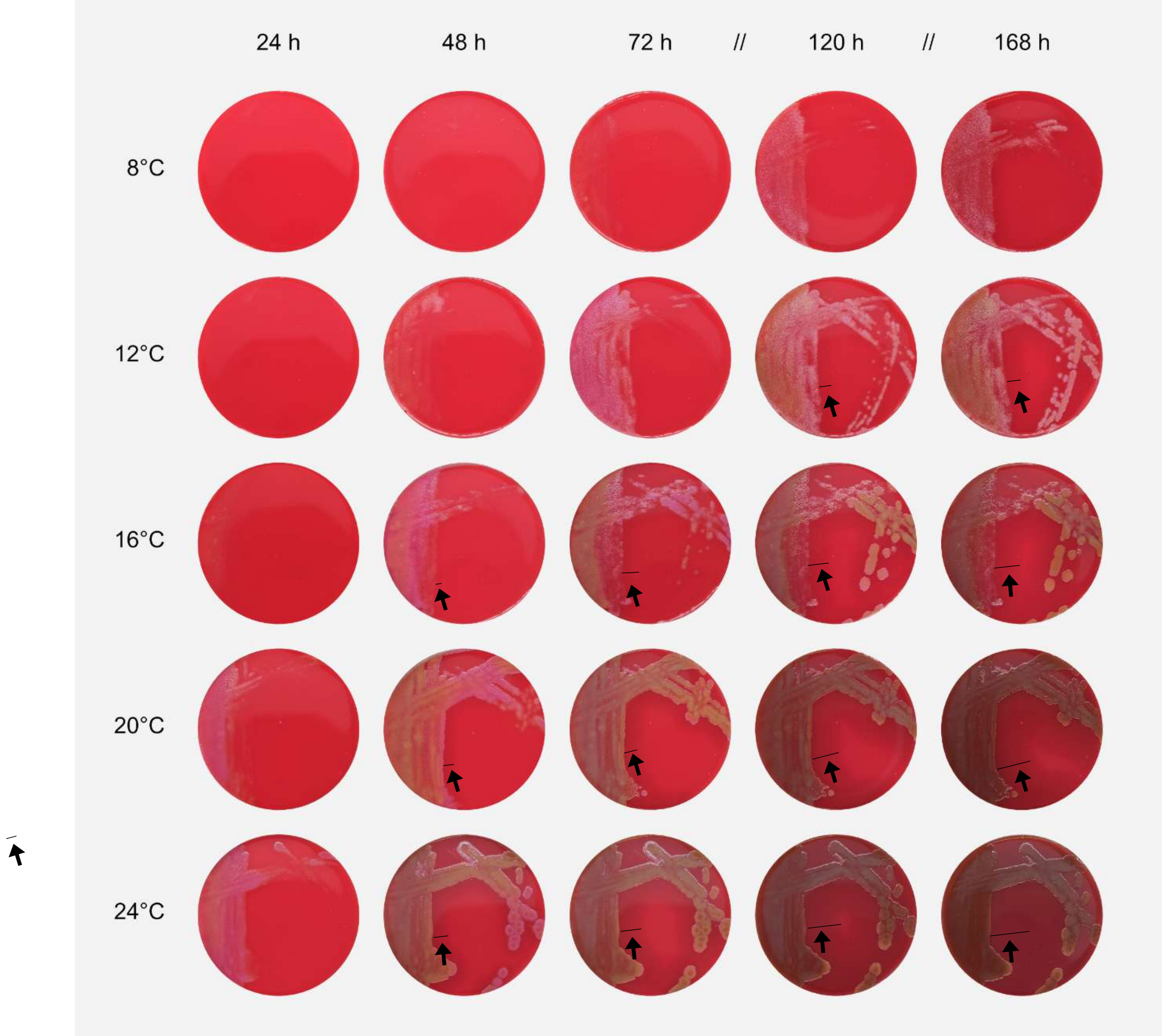

C. Growth of NS17on BAMA agar plate

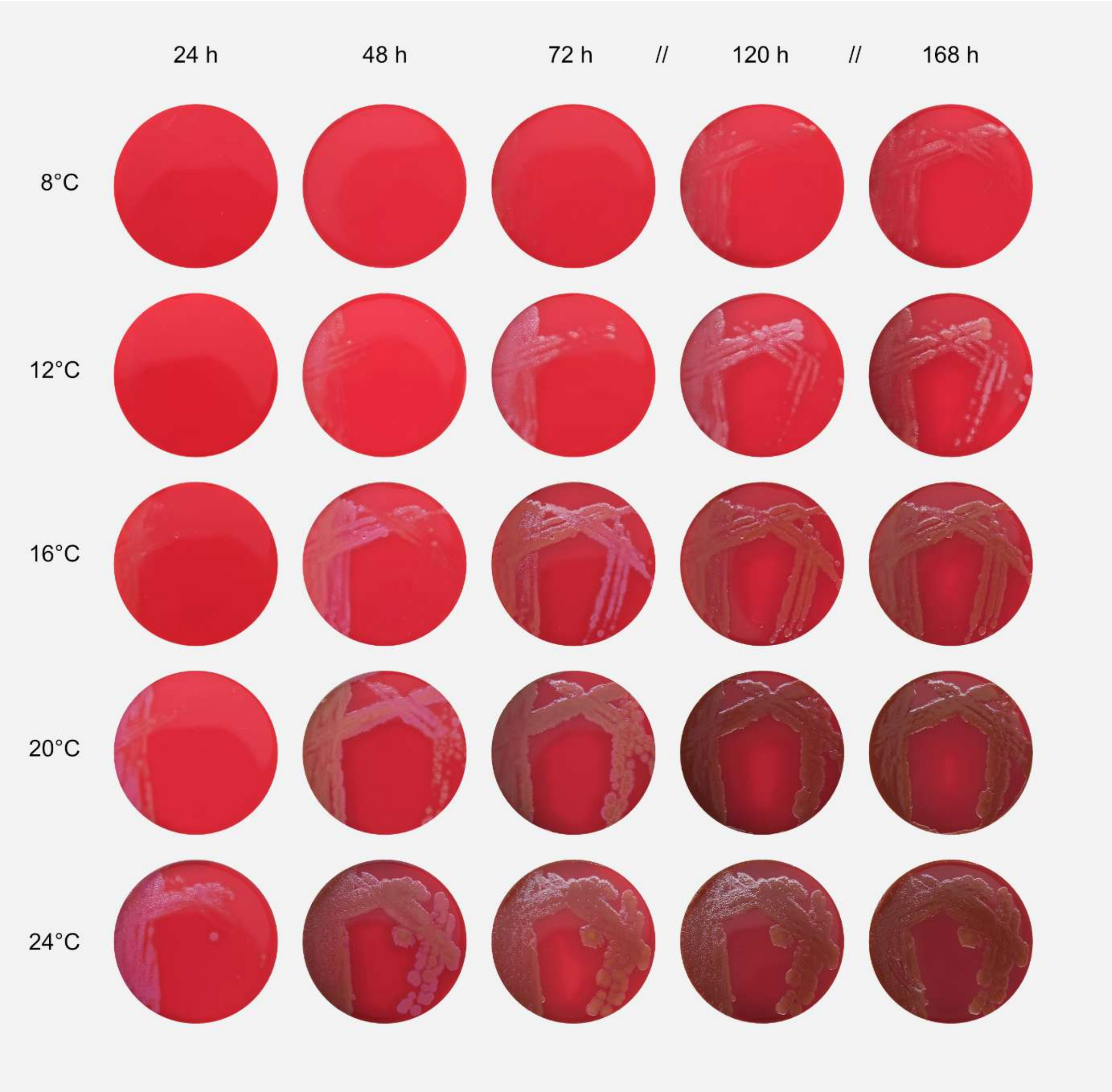

D. Growth of NH18 on BAMA agar plate

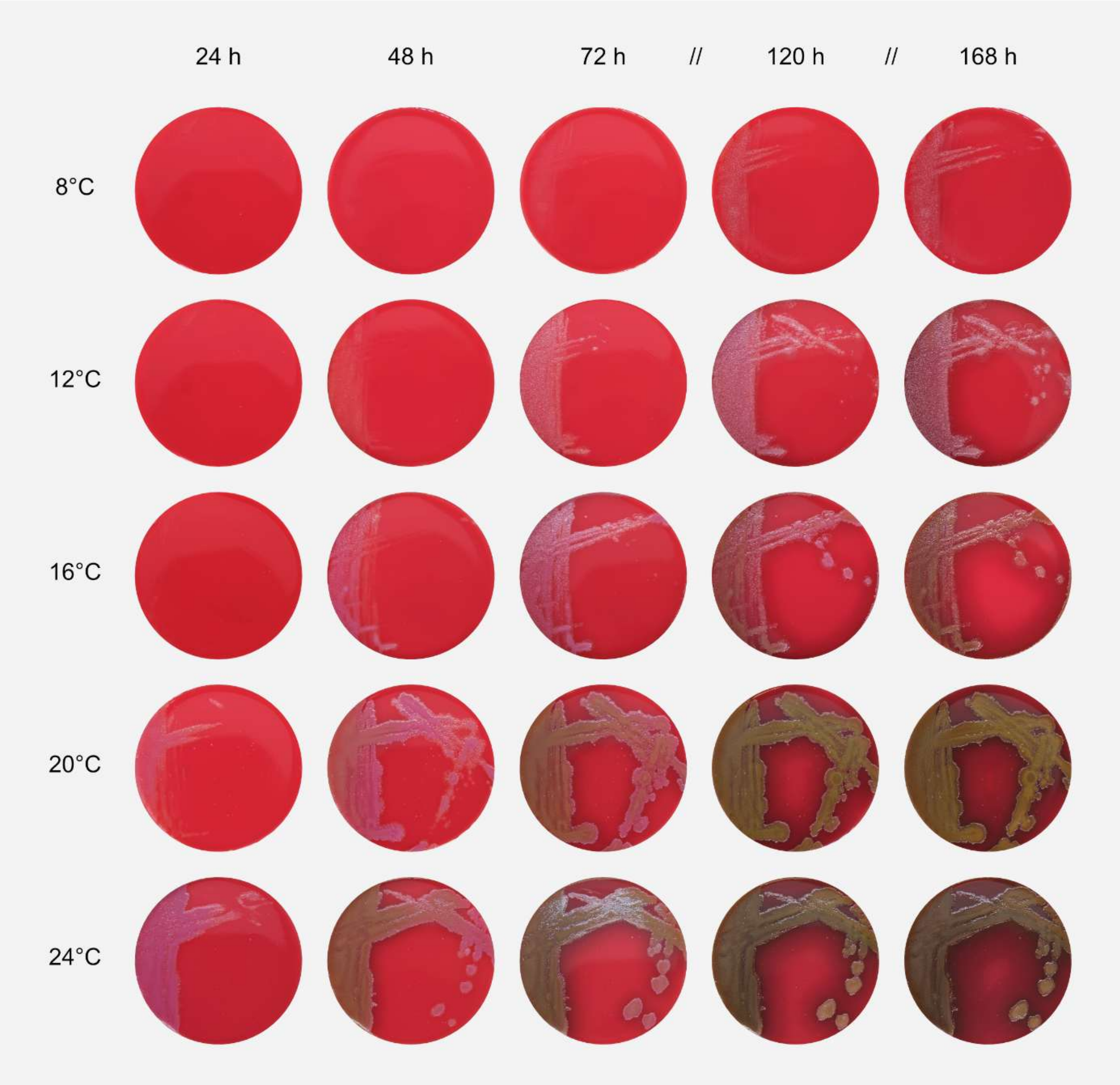

Supplement: Supplementary file 4 [file pone.0361557.s006.pdf]
